# Identification of angiogenesis‐related genes signature for predicting survival and its regulatory network in glioblastoma

**DOI:** 10.1002/cam4.6316

**Published:** 2023-07-11

**Authors:** Zhiping Wan, Xiaokun Zuo, Siqiao Wang, Lei Zhou, Xiaojing Wen, Ying Yao, Jiefang Song, Juan Gu, Zhimin Wang, Ran Liu, Chun Luo

**Affiliations:** ^1^ Department of Neurosurgery, Tongji Hospital, School of Medicine Tongji University Shanghai China; ^2^ Division of Spine, Department of Orthopedics, Tongji Hospital, School of Medicine Tongji University Shanghai China; ^3^ Department of Orthopedics Jinxian County People's Hospital Nanchang China; ^4^ Department of Infection Jinxian County People's Hospital Nanchang China; ^5^ Department of Operating Room, Tongji Hospital, School of Medicine Tongji University Shanghai China; ^6^ Department of Emergency, Ruijin Hospital Luwan Branch Shanghai Jiaotong University School of Medicine Shanghai China; ^7^ The Medical School of Zhengzhou University Zhengzhou City People's Republic of China

**Keywords:** angiogenesis, glioblastoma, multi‐omics, prognostic biomarkers, regulatory network

## Abstract

Glioblastoma (GBM) is notorious for malignant neovascularization that contributes to undesirable outcome. However, its mechanisms remain unclear. This study aimed to identify prognostic angiogenesis‐related genes and the potential regulatory mechanisms in GBM. RNA‐sequencing data of 173 GBM patients were obtained from the Cancer Genome Atlas (TCGA) database for screening differentially expressed genes (DEGs), differentially transcription factors (DETFs), and reverse phase protein array (RPPA) chips. Differentially expressed genes from angiogenesis‐related gene set were extracted for univariate Cox regression analysis to identify prognostic differentially expressed angiogenesis‐related genes (PDEARGs). A risk predicting model was constructed based on 9 PDEARGs, namely MARK1, ITGA5, NMD3, HEY1, COL6A1, DKK3, SERPINA5, NRP1, PLK2, ANXA1, SLIT2, and PDPN. Glioblastoma patients were stratified into high‐risk and low‐risk groups according to their risk scores. GSEA and GSVA were applied to explore the possible underlying GBM angiogenesis‐related pathways. CIBERSORT was employed to identify immune infiltrates in GBM. The Pearson's correlation analysis was performed to evaluate the correlations among DETFs, PDEARGs, immune cells/functions, RPPA chips, and pathways. A regulatory network centered by three PDEARGs (ANXA1, COL6A1, and PDPN) was constructed to show the potential regulatory mechanisms. External cohort of 95 GBM patients by immunohistochemistry (IHC) assay demonstrated that ANXA1, COL6A1, and PDPN were significantly upregulated in tumor tissues of high‐risk GBM patients. Single‐cell RNA sequencing also validated malignant cells expressed high levels of the ANXA1, COL6A1, PDPN, and key DETF (WWTR1). Our PDEARG‐based risk prediction model and regulatory network identified prognostic biomarkers and provided valuable insight into future studies on angiogenesis in GBM.

## Graphical abstract

In this study, we have constructed a regulatory network based on angiogenesis‐related gene signature and identified a signaling pathway, WWTR1 (TAZ)‐ANXA1, COL6A1, and PDPN‐EMT, which may provide new therapeutic strategies by utilizing the signaling axis in tumor angiogenesis we proposed for GBM patients.

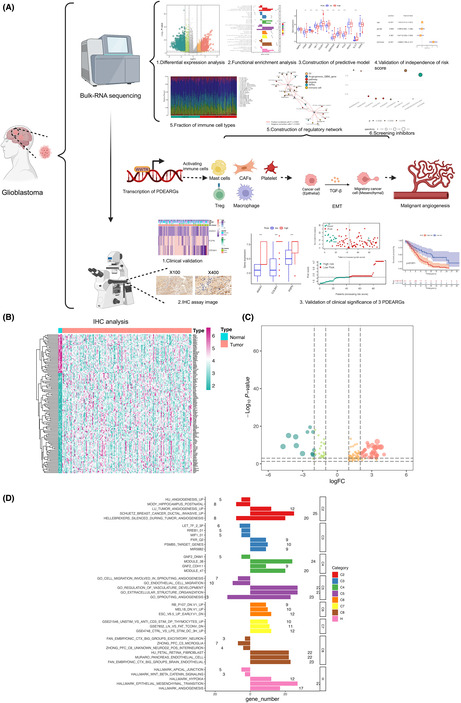

## 1 INTRODUCTION

As the most intractable and malignant grade of glioma, GBM is considered to develop from either dedifferentiated glial cells or neuroglial progenitor cells.^1^, ^2^, ^3^ Advances in research on molecular and cellular pathology contributing to malignant transformation have offered effective handles to GBM, yet the patients have not profited much. Glioblastoma has a median survival of 12–18 months, remaining undesirable for nearly half of the past century, in spite of new‐standard therapy strategies.^4^, ^5^ Hence, the prognosis for GBM patients remains unsatisfactory, and new predictive biomarkers and therapeutic targets are urgently needed.

Angiogenesis has been increasingly mentioned as a hallmark of the progression in GBM. High metabolic demands of GBM create hypoxic areas and require newly generated vessels to contain oxygen and nutrients to meet the need of GBM tumor growth and facilitate the migration of GBM cells. Most importantly, more than 90% of glioma recurrences are found within 2 cm of the focal area and prone to malignant progression,^6^ in which angiogenesis may play a leading role. Consequently, more in‐depth studies on alternative transcription factors (TFs), genes, and molecular pathways regulating angiogenesis may help identify agreeable targets for GBM patients' treatment. A myriad of studies have revealed key receptors and factors for angiogenesis in GBM, which serve as diagnostic markers and therapeutic targets, such as vascular epithelial growth factor (VEGF), hypoxia‐inducible gene 2 (HIG2), and angiopoietins (Angs).^7^ Yet, combination treatment targeting these markers such as VEGF has meager efficacy and inexorable cytotoxic effect due to acquired resistance,^8^ which demonstrates the need for more studies on novel hub molecules and signaling pathways fueling malignant vascularization in GBM given its peculiarity.^9^ To our knowledge, previous studies have not been focused on the transcription factor–angiogenesis‐related gene–pathway regulatory networks and prognostic models.

Here, transcriptome sequencing data of GBM patients were evaluated, and the correlation of expression of angiogenesis‐related genes and clinical outcomes was explored. Single‐cell RNA sequencing data and immunohistochemistry (IHC) was used to validate the expression of core angiogenesis‐related genes. Connectivity map database was employed to select potential chemical agents targeting angiogenesis‐related genes.

## 2 METHODS AND MATERIAL

### 2.1 Data extraction and participants

RNA sequencing (RNA‐seq) data and 173 samples obtained from patients with GBM were collected from TCGA (https://portal.gdc.cancer.gov/). R packages of “affy” and “impute” in R/Bioconductor software were utilized for RNA‐seq data processing. Elaborate sample information of demographic feature (gender, age, and ethnicity), clinical stages, survival end point (cutting‐edge, days to death and days to the last follow‐up) even histopathology were also retrieved. Samples with imprecise clinical information was excluded. 318 tumor‐flag TFs and its expression profiles were collected from Cistrome database (http://cistrome.org/).^10^ Additionally, the ImmPort (immport.org/home) database provided us with immune‐correlated genes and immune cells.^11^ The schematic diagram of our study was created with BioRender.com (https://biorender.com/).

From 2018 to 2021, we obtained tumor tissue samples and clinical information of patients with GBM, including 95 GBM tissues, from the Neurosurgery Ward of the Shanghai Tongji Hospital affiliated to Tongji University. We state that our study was granted by the Ethics Committee of Tongji Hospital affiliated to Tongji University (No. SBKT‐2022‐053), and we conducted the research in strict accordance with its regulations. The inclusion criteria of our study were set to cover candidates as: (1) patients were aged at 18 or over, diagnosed with GBM pathologically, and have been surgical treated in our center; (2) patients' clinical information for this study were completed. The exclusion criteria were set to be precise as: (1) patients were diagnosed with other malignancies; (2) patients' pathological sections were missing; and (3) patients were untraceable to follow‐up.

### 2.2 DEGs identification and function enrichment analysis

To analyze the expression profile, the log_2_ (fold change [FC]), false discovery rate (FDR), *t* value and *p* value of each detected gene between 168 GBM tumor samples and 5 para‐tumor tissue samples were calculated by R package “limma.” Differentially expressed genes were defined from genes of absolute value log_2_ (FC) > 1 and *p* < 0.05. Angiogenesis‐related genes (ARGs) info were obtained from Molecular Signature Database (MSigDB, https://www.gsea‐msigdb.org/gsea/msigdb/) and members from angiogenesis‐related gene set. Likewise, differentially expressed ARGs (DEARGs) were determined using “limma” package, based on the thresholds of |log2 (FC)| > 1.0 and *p* < 0.05. Gene set enrichment analysis (GSEA) and over‐representation analysis (ORA) were conducted based on MSigDB and Gene Ontology (GO) database, to identify potential biological processes involving the DEARGs. The biological function of the overlap DEARGs was interpreted. There was statistical significance in the *p* value (<0.05).

### 2.3 Risk signature construction and assessment

To ascertain the prognostic indicators from DEARGs, univariate Cox regression analysis was conducted by the expression profiles and clinical outcomes of each sufferer with GBM. Specifically, DEARGs with *p* value <0.05 were adopted as prognostic DEARGs (PDEARGs). Forest plot was generated to describe hazard ratio (HR) and *p* value of each PDEARGs.

### 2.4 A risk prediction model was established based on the PDEARGs

We then set a loop by R programming language where the GBM patients were partitioned into train and test cohort by ratio of 3:2 randomly. In each cycle, by using R package “glmnet”,^12^ aforementioned PDEARGs were included in Lasso regression analysis to further evade overfitting risk by removing the strong collinearity PDEARGs. To build a risk‐prediction model at first, multivariate Cox regression analysis was conducted by running R package “survival.” We used the “predict” function of R to quantize the risk score (RS), and the score is calculated as: RS = gene_1_ expression *β_1_ + gene_2_ expression *β_2_ + … + gene_n_ expression *β_n_ (*β*:regression coefficient of the genes in the model).

Risk score of each GBM patient was obtained, and directly displayed by vascular content in Hematoxylin–Eosin (HE) staining and Ki‐67 in clinicopathological histochemistry. According to the cutoff value of RS, these GBM patients were separated into the group of low‐ or high‐risk. Package “survminer” was utilized to determine the optimal cutoff for risk‐grouping of different cohorts, respectively. The survival of each individual was displayed by risk curve and scatter plot. Differences in survival were identified by Kaplan–Meier survival analysis between low‐ and high‐risk group. Receiver operating characteristic (ROC) curve, along with its area under the curve (AUC), both were investigated respectively to make sure the accuracy of the risk‐predicting model by package “survivalROC.” Moreover, this loop would break when *p* value <0.01 and AUC >0.85 in the train cohort, along with *p* < 0.05 and AUC >0.80 in the test cohort.

Univariate and multivariate analyses of Cox regression were conducted to distinguish whether RS were an independent factor along with other clinical features including age, race, and gender. Furthermore, to reveal the function of PDEARGs, we made gene set enrichment analysis (GSEA, http://software.broadinstitute.org/gsea/index.jsp)^13^ verify the RERTR of previously identified biological processes (GO, KEGG, and hallmark pathways) in the ranked PDEARGs between both risks group.

### 2.5 Validation on genomic level

Frequency of copy number variations (CNVs) of PDEARGs was calculated and the loci of these genes on chromosomes were visualized. The spearman correlation analysis was adopted to analyze the relationship between the tumor mutation burden (TMB) and the risk score. Data concerning the number of mutated genes in GBM samples were retrieved and the waterfall plot was created to describe the top 20 mutated genes in low‐ and high‐risk groups.

### 2.6 Identification of differentially expressed TFs (DETFs) co‐expressed with PDEARGs

To explore the DETFs, we further applied the “limma” packages in R to process TFs' expression profiles between 168 GBM tumor samples and five adjacent normal tissue samples. TFs were regarded as DETFs with its absolute value of log_2_ (fold change) >1 and in the meantime *p* < 0.05. Pearson's correlation analysis were conducted to confirm co‐expression of DETFs and PDEARGs. Only correlation meets Pearson's correlation coefficient (*R*) >0.60 and *p* < 0.05 were adopted for subsequent analyses.

### 2.7 Identification of immune infiltration patterns and immune function in GBM angiogenesis

Immune cell, contributing to angiogenesis in patients with GBM, were identified by estimating relative subsets of RNA transcripts (CIBERSORT) algorithm.^14^ Infiltration levels of 22 immune cell types were acquired from the CIBERSORT (https://cibersort.stanford.edu/). After removing samples without statistical significance, we constructed bar plots to reveal the distribution and classification of these cell subtypes in GBM patients within the both risk groups. Single‐sample gene‐set enrichment analysis (shorthand as “ssGSEA”) sooner was carried out aiming at ascertaining the immune function score per each sample based on their expression patterns by using 29 immune datasets, namely immune cell subtypes, immune‐related activities, and pathways.^15^ Comparisons were performed between immune cell subtypes and immune function scores in the low‐ and high‐risk groups. Our members adopted K‐M survival analysis to dig out the correlation between immune cell types as well as immunity functions and survival possibility in GBM patients. Pearson's correlation analysis was performed to identify immune cell subtypes and immunity functions correlated to PDEARGs. PDEARG‐immune cell pairs with absolute value of *R* > 0.20 and *p* < 0.05 and PDEARG‐immune function pairs with *R* > 0.48 and *p* value <0.05 were considered closely correlated.

### 2.8 External validation by multiple databases

A total of 7202 GBM samples and their profiling in terms of immune dysfunction and exclusion (TIDE), Dysfunction, DKK3, SLIT2, SERPINA5, Merck18, PLK2, and NRP1 were retrieved from the TIDE database (http://tide.dfci.harvard.edu/login/)^16^ to test the robustness of the prediction model and the violin plots were constructed to illustrate the difference of aforementioned biological behavior (TIDE, Dysfunction, DKK3, SLIT2, SERPINA5, Merck18, PLK2, and NRP1) between the low‐ and high‐ risk group. ROC curve of risk prediction model was utilized to predict the survival of GBM patients. Moreover, we compared the AUC of ROC curve of our prediction model and those from TIDE and TIS database to demonstrate the superiority of our prediction model.

### 2.9 Construction of regulatory network and identification of specific inhibitors

Gene set variation analysis (namely “GSVA”)^15^ together with R package “limma” were utilized to identify hallmark signaling pathways expressed differentially between the tumor samples and adjacent noncancerous samples. In addition, we tested the correlation between PDEARGs and hallmark signaling pathways, PDEARGs and DETFs, PDEARGs and immunity functions, PDEARGs and RPPA chips, PDEARGs and immune cell subtypes in succession by Pearson's correlation analysis. Only PDEARGs with absolute value of *R* > 0.52 and *p* < 0.05 (in PDEARG‐hallmark signaling pathway pairs), absolute value of *R* > 0.60 and *p* < 0.05 (in PDEARG‐DETF pairs), absolute value of *R* > 0.48 and *p* < 0.05 (in PDEARG‐immunity function pairs), absolute value of *R* > 0.50 and *p* < 0.05 (in PDEARG‐RPPA chip pairs), absolute value of *R* > 0.20 and *p* value <0.05 (in PDEARG‐immunity cell pairs) were accounted as pivotal genes in the regulatory network. Based on the abovementioned correlation pairs, a regulatory network composed of DETFs, PDEARGs, signaling pathways, immune cell types, immune functions, and RPPA chips was established, and the regulatory network was visualized by using Cytoscape 3.6.

### 2.10 Screening of small molecule drugs

To predetermine the small molecular drugs which could attenuate or reverse the effect brought by identified PDEARGs and DETFs, the Connectivity map (CMap) database^17^ was utilized to explore the putative molecular drugs for the treatment of GBM. CMap compares gene signatures with whole genomic expression profiles of multiple cell lines, when treated with more than 1000 compounds that were almost all approved by the United States FDA. First, we have constructed a genes signature based on the identified PDEARGs and DETFs (|log2FC| ≥ 1, FDR < 0.05) in this study. Second, we uploaded this signature into the CMap database.

Similarity of the query to each of the gene signatures were represented by connectivity score (CS) of each agent, ranging from −1 to 1, was calculated. Specifically, negative CS indicated that the drug may reverse input results. Subsequently, we identified small molecule drugs with negative CSs, which demonstrated the therapeutic potent for GBM.

### 2.11 Validation of the PDEARG signature in the independent validation cohort

We further accessed the PDEARG signature in the independent validation cohort collected from our own institution. Only five GBM patients in the primary cohort were excluded due to loss to follow‐up. The whole cohort (*n* = 95) was divided into a train set (*n* = 60) and test set (*n* = 35) at a ratio of 3:2 randomly. We conducted Univariate LASSO regression, multiple Cox regression analysis, and Cox regression analysis to evaluate the clinical application value of PDEARGs in predicting the overall survival (OS) of the patients, and to rebuild a risk prediction model. Based on the median value of the risk score, all GBM patients in the train set were divided into low‐ and high‐risk groups, respectively. K–M curves, used for OS evaluation of the two groups were constructed to assess the accuracy of the PDEARG signature in the internal train group. We further verified the results in the internal test group.

### 2.12 Construction and evaluation of the Cox proportional hazard models

Upon the GBM patient's clinical data and RS, independent prognostic factors were estimated utilizing univariate, multivariate Cox regression analyses. We assessed the proportional hazard assumption by Schoenfeld residuals with R package “RMS.” Likelihood ratio tests were conducted to evaluate the Cox proportional hazard models.

### 2.13 Immunohistochemistry (IHC) and Hematoxylin–Eosin (HE) staining

Tumor tissue sections pathological diagnosed as GBM, were collected from the Neurosurgery Ward of the Shanghai Tongji Hospital after the consent of 95 GBM patients was obtained.

### 2.14 Immunohistochemistry (IHC)

After been blocked with endogenous peroxide and protein, the tissue sections were incubated with diluted specific anti‐ANXA1 (ab214486), anti‐COL6A1 (ab6588), and PDPN (ab10288) at 4°C for 12 h. Then, the sections were incubated using the secondary antibody at 37°C for 1 h. Next, we use 3,3‐diaminobenzidine solution to stain those sections for 3 min and counterstained using hematoxylin. Eventually, the slices were observed, analyzed, and photographed by microscope.

### 2.15 Hematoxylin–Eosin (HE) staining

After dewaxing, the tissue sections were stained with Hematoxylin and Eosin solution (G1003, Servicebio). Then, sections were subjected to dehydrate. Finally, we observed their morphology, malignancy degree, and vascular content with microscope, captured images and conducted analysis when the GBM sections stained successfully.

### 2.16 ATAC‐seq data analysis

Assay for Transposase‐Accessible Chromatin sequencing (ATAC‐seq) was used for studying the chromatin‐accessibility of hub genes.^18^ We downloaded the Bigwig files from TCGA ATAC‐seq program (https://gdc.cancer.gov/about‐data/publications/ATACseq‐AWG) and visualized the result through using R package of Gviz.^19^

### 2.17 Single‐cell transcriptome analysis

As single‐cell RNA sequencing (scRNA‐seq) assays continues to develop and data availability increases to expand, scRNA‐seq datasets have been widely used. Furthermore, the scRNA‐seq on 3589 cells in a cohort of four GBM patients^20^ was downloaded from the Single Cell Expression Atlas (accession number: GSE84465) at https://www.ebi.ac.uk/gxa/sc/experiments/E‐GEOD‐84465/results.

First, we generated a Single‐Cell‐Experiment object by loading 10× single‐cell raw matrix into R statistical programming software (v4.0.2) through Seurat package (v4.0.1). The matrix of data was then normalized, rescaled and filtered via quality control (QC). Second, the top 20 components of principal components analysis (PCA), as well as supplementary t‐SNE analysis, were conducted (perplexity = 50). We conduct clustering of similar single cell groups on the two‐dimensional (2D) t‐distributed stochastic neighbor embedding (tSNE) space with k‐means and by R package “stats.” Feature plot was utilized to visualize specific gene expressions from single‐cell data.

### 2.18 Single‐cell functional analysis by CancerSEA database

We further investigated the correlations of gene expression levels of the identified differentially expressed angiogenesis‐related genes with 14 functional states in multiple cancer types, especially in GBM, including apoptosis, angiogenesis, differentiation, cell cycle, DNA repair, DNA damage, EMT, inflammation, hypoxia, proliferation, invasion, metastasis, stemness, and quiescence, by utilizing the CancerSEA database (http://biocc.hrbmu.edu.cn/CancerSEA/). The threshold of the aforementioned genes related to each cancer functional status was established as a threshold value of |*r*| > 0.3 and a discrimination significance (*p* < 0.05).

### 2.19 Chinese Glioma Genome Atlas (CGGA) data validation

The data of 389 GBM patients were obtained from the CGGA (www.cgga.org.cn) database. Because of incomplete information, 100 GBM patients were excluded from this study, and we only investigated the data of the remaining 289 GBM patients. Kaplan–Meier curves were constructed to validate the potential relationship between the OS of GBM patients and the expression level of the identified differentially expressed angiogenesis‐related genes.

### 2.20 Statistical analysis

All the data were visualized and analyzed using R software (R Foundation for Statistical Computing, Vienna, Austria, 3.6.1) involving packages “limma,” “clusterProfiler,” “msigdbr,” etc. (see in the Data S1). Comparisons of the two groups were conducted by utilizing Mann–Whitney tests or Student's *t*‐tests. To evaluate the discrepancy among multiple groups, variance was analyzed by One‐way methods. Statistical significance was described and replaced (RE) as follows: not significant RE ns; *p* < 0.05 RE *; *p* < 0.01 RE **, and *p* < 0.001 RE ***.

## 3 RESULTS

### 3.1 Identification of DEGs and function enrichment analysis

**FIGURE 1:**
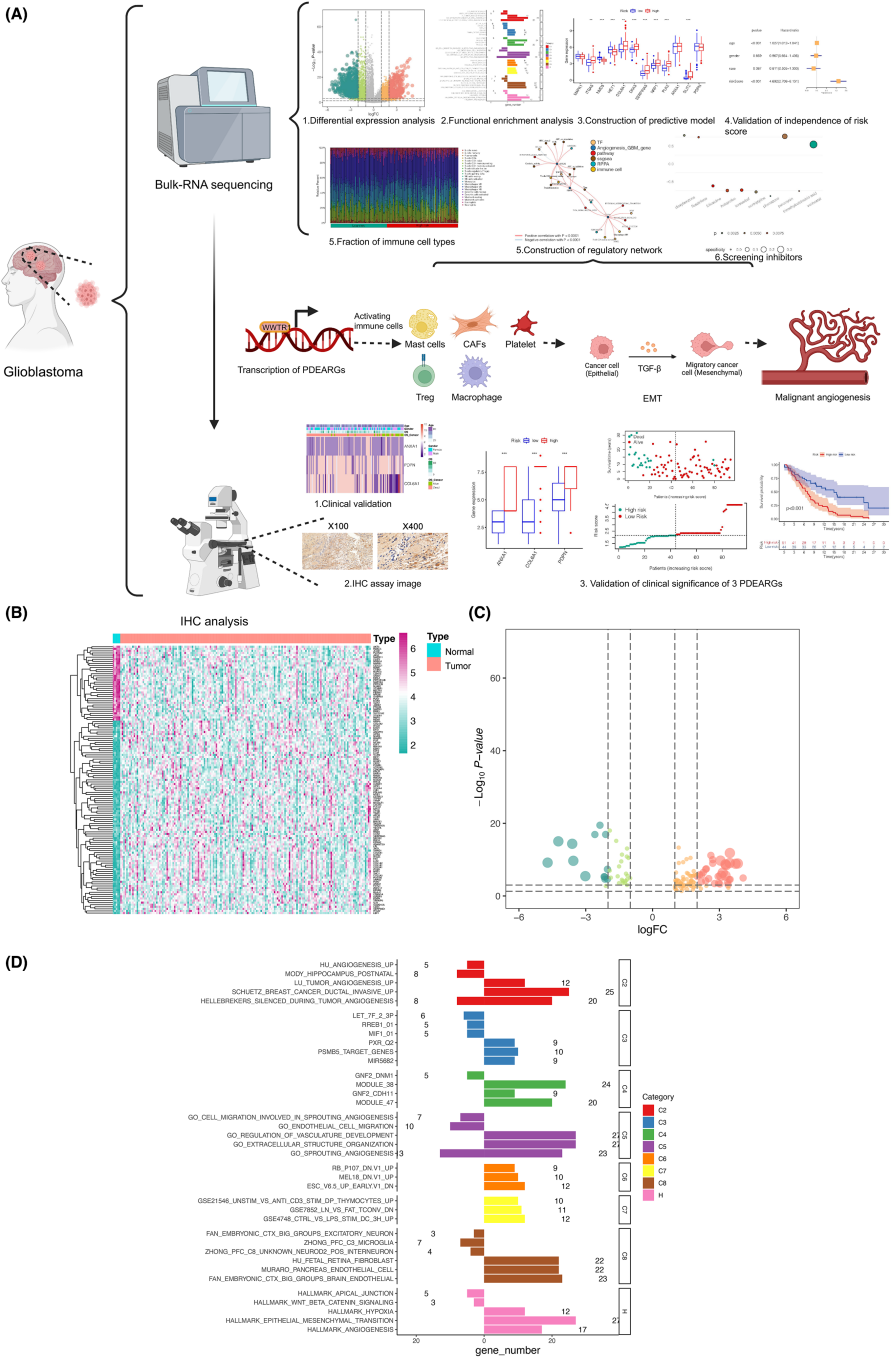
Identification of differentially expressed angiogenesis‐related genes and enrichment analysis. (A) The schematic diagram of our study. (B) Heatmap plot showing expression level of 129 differentially expressed angiogenesis‐related genes between tumor and normal tissues in glioblastoma (GBM). (C) Volcano plot displaying ‐log10 (*p*‐value) and log2 (fold change [FC]) of the differentially expressed angiogenesis‐related genes, where red represented up‐regulation while green represented down‐regulation. (D) Bar plot showing the results of over representation analysis (ORA). It indicated that differentially expressed angiogenesis‐related genes were closely related to certain signaling pathways relevant to tumor invasion and angiogenesis.

The procedures of our study were illustrated in Figure 1A and Figure S1. A total of 173 samples and their RNA sequencing data from GBM patients including 168 tumor samples and 5 peritumoral tissue samples were retrieved from TCGA database. As a result, 4722 DEGs between tumor tissues and normal ones were identified (Figure S2A,B). A total of 412 genes involved in tumor angiogenesis were downloaded and 129 DEARGs were identified based on the same thresholds (Figure 1B,C). Additionally, 60 out of 318 cancer‐related TFs were recognized as differentially expressed (Figure S2C,D).

Then, the 129 DEARGs, including 36 downregulated genes and 93 upregulated genes, were extracted for ORA and a set of biological processes were unveiled (Figure 1D). It revealed that DEARGs were closely associated with certain signaling pathways relevant to tumor invasion and angiogenesis, such as Schuetz breast cancer ductal invasive up, GO regulation of vasculature development, GO extracellular structure organization, GO sprouting angiogenesis, and hallmark epithelial mesenchymal transition (Figure 1D; Figure S2E).

### 3.2 Construction of the PDEARG signature

Given the clinical application potential of the DEARGs in GBM, we attempted to identify the prognostic and diagnostic effects of DEARGs. First, we paired the DEARG expression profiles with their clinical information to build a complete risk prediction model by inputting profile consisting of 160 GBM patients. Notably, to reduce the bias to the minimum, the filtered TCGA‐GBM patients were divided randomly into the train set (*n* = 96; 60%) and test set (*n* = 64; 40%). Second, to obtain PDEARGs that were closely correlated with prognosis of patients with GBM, univariate Cox regression analysis were conducted in the TCGA‐GBM train set. The results showed that 27 of these DEARGs were closely related to the overall survival (OS) of GBM patients, which were defined as PDEARGs (*p* < 0.05; Figure S3).

### 3.3 Construction and evaluation of the prediction model

**FIGURE 2:**
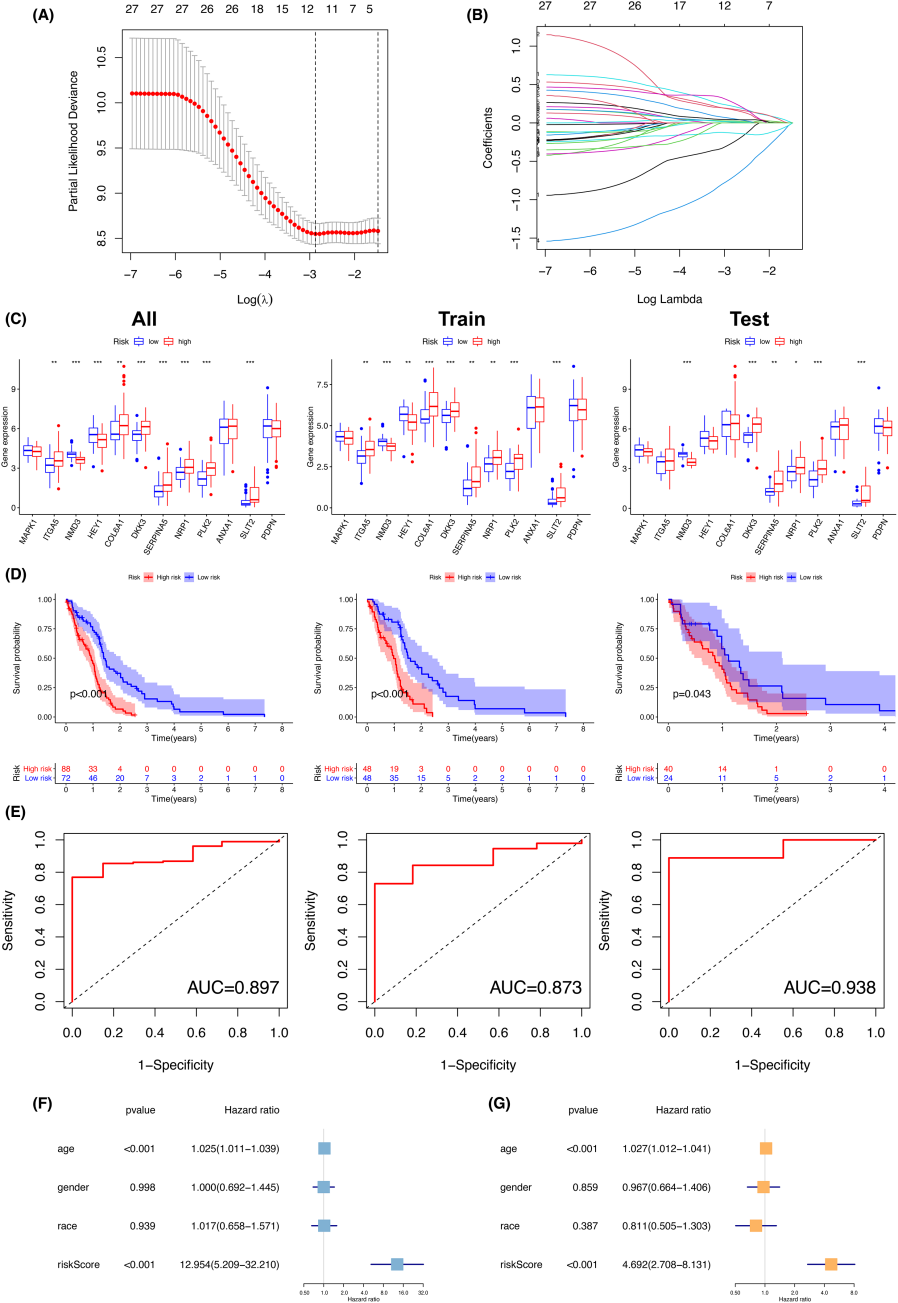
Screening the prognostic differentially expressed angiogenesis‐related genes and validation. (A, B) Lasso plot and lambda plot from the Lasso regression analysis. (C) Box plots showing the difference of 9 prognostic differentially expressed angiogenesis‐related genes between low‐ and high‐risk group. (D) Kaplan–Meier survival analysis displaying difference of survival probability between low‐ and high‐risk group (*p* < 0.001 in all set, *p* < 0.001 in train set, and *p* < 0.05 in test set). The mortality rate in the high‐risk group was significantly higher than that in the low‐risk group. (E) Area under the curve (AUC) of receiver operating characteristic (ROC) curve demonstrating the robustness of the predictive model (AUC = 0.897 in all set, AUC = 0.873 in train set, and AUC = 0.938 in test set). (F, G) Univariate (HR = 12.954, 95% CI (5.209–32.210), *p* < 0.001) and multivariate (HR = 4.692, 95% CI (2.708–8.131), *p* < 0.001) Cox regression analysis confirming risk score to be an independent prognostic factor.

To prevent the model from being over‐fitting, we applied the Lasso regression analysis to obtain the significant PDEARGs (Figure 2A,B). Eventually, a 12‐PDEARG prognostic signature consisting of MAPK1, ITGA5, NMD3, HEY1, COL6A1, DKK3, SERPINA5, NRP1, PLK2, ANXA1, SLIT2, PDPN was employed to calculate the risk score of GBM samples. By employing the coefficients obtained from the Lasso regression, we obtained the risk score for each GBM patient. The formula used for calculating risk score was as follows: risk score = risk score = (0.681 * exp of MAPK1) + (1.372 * exp of ITGA5) + (0.517 * exp of NMD3) + (0.762 * exp of HEY1) + (1.205 * exp of COL6A1) + (1.489 * exp of DKK3) + (1.214 * exp of SERPINA5) + (1.372 * exp of NRP1) + (1.380 * exp of PLK2) + (1.176 * exp of ANXA1) + (1.380 * exp of SLIT2) + (1.172 * exp of PDPN). Based on the optimal cutoff of risk score, GBM patients were categorized into high‐ and low‐risk groups.

The performance of the risk prediction model was evaluated in TCGA‐GBM train and test sets, as well as in all sets (160 GBM patients). Expression level difference of 12 PDEARGs between high‐ and low‐risk groups was demonstrated in box plots (Figure 2C). All sets showed excellent survival prediction capability. The difference of survival possibility between the low‐ and high‐risk groups was significant in the Kaplan–Meier survival analysis (*p* < 0.001 in all set, *p* < 0.001 in train set, and *p* < 0.05 in test set) and the mortality rate in the high‐risk group was higher than that in the low‐risk group (Figure 2D). The AUC of the 12‐PDEARG prognostic signature (AUC = 0.897 in all set, AUC = 0.873 in train set, and AUC = 0.938 in test set) illustrated the robustness and reliability of our risk prediction model (Figure 2E). Additionally, PDEARGs were proved to be an independent factor along with age, gender, and race in both univariate (HR = 12.954, 95% CI (5.209–32.210), *p* < 0.001) and multivariate Cox regression analysis (HR = 4.692, 95% CI (2.708–8.131), *p* < 0.001) (Figure 2F,G).

**FIGURE 3:**
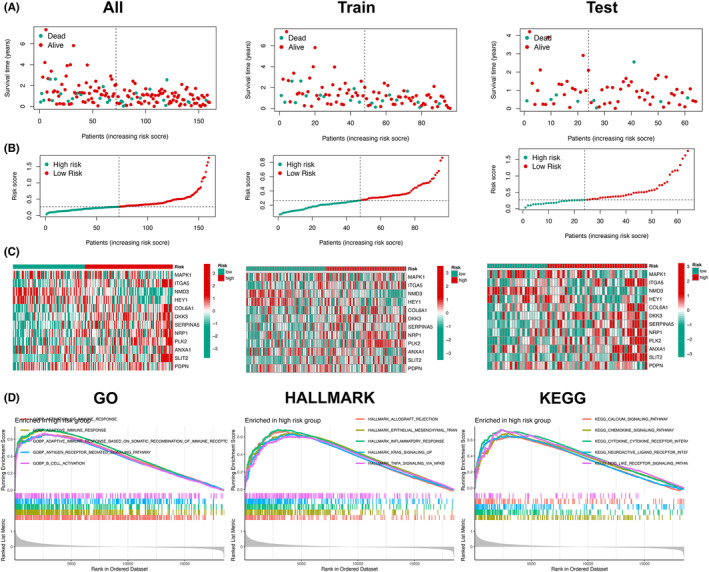
Prognostic model for patients with glioblastoma (GBM). (A) The scatter plot showing the survival time of each patient. (B) The risk curve chart displaying the risk score of each patient. (C) The heatmap plot showing the expression level of 12 prognostic differentially expressed angiogenesis‐related genes in patients from low‐ and high‐risk groups. (D) Result of gene set enrichment analysis (GSEA) in gene ontology (GO), hallmark and Kyoto encyclopedia of genes and genomes (KEGG) databases. The prognostic differentially expressed angiogenesis‐related genes enriched in high‐risk patients were associated with immune or inflammation‐related pathways.

Moreover, the scatter plot and risk curve displayed the notable differences in survival status and risk score of GBM patients between low‐ and high‐risk groups (Figure 3A,B). The heatmap showed the significantly differential expression level of the 12 PDEARGs in patients from low‐ and high‐risk groups (Figure 3C). GSEA was performed to fully unveil the implicit mechanisms that drive angiogenesis within the tumors, thus contributing to undesirable clinical outcomes in patients with GBM. Our findings indicated that the gene sets enriched in high‐risk patients were associated with immune or inflammation‐related pathways, including immune somatic recombination receptors for GOBP adaptive immune response, KEGG cytokine‐cytokine receptor interaction and hallmark inflammatory response (Figure 3D).

### 3.4 Validation of the 12‐PDEARG signature on genomics level

**FIGURE 4:**
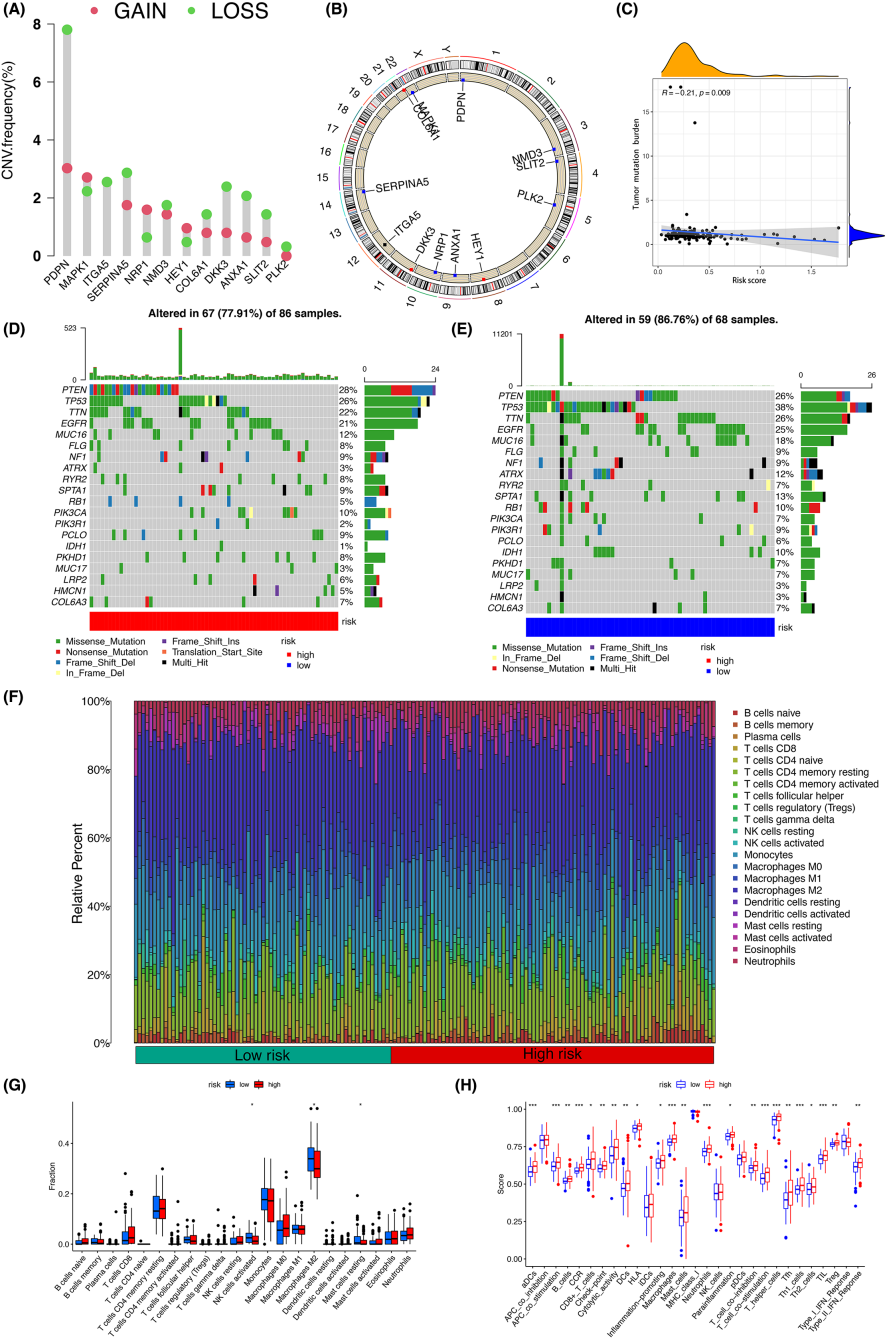
Validation on genomics level and identification of immune infiltration. (A) The bar plot showing the frequency of copy number variation (CNV) of 12 prognostic differentially expressed angiogenesis‐related genes. Podoplanin (PDPN) showed the highest frequency of gene gain and loss. (B) Circle plot visualizing the loci of 12 prognostic differentially expressed angiogenesis‐related genes on the chromosomes. (C) Spearman correlation analysis revealing the negative relationship between tumor mutation burden (TMB) and risk score (*R* = −0.21, *p* = 0.009). (D, E) Waterfall plots showing the top 20 mutated genes in low‐ and high‐risk groups. (F) Bar plot displaying the abundances of 22 immune cell types in glioblastoma patients from low‐ and high‐risk groups. (G, H) Box plots revealing difference of immune cells and immune functions between low‐ and high‐risk groups. NK cells activated (*p* < 0.05), Macrophages M2 (*p* < 0.05), and Mast cells resting (*p* < 0.05) were identified to be significantly increased in low‐risk group.

To confirm the reliability of the prediction model on genomics level, the copy number variant (CNV) frequency of 12 genes was explored and PDPN showed the highest frequency of gene gain and loss (Figure 4A). The specific location of these PDEARGs on chromosome was also shown in the circle plot (Figure 4B). To unveil the correlation of the risk score and tumor mutation burden (TMB), the spearman correlation analysis was performed and TMB turned out to be negatively correlated to the risk score (*R* = −0.21, *p* = 0.009) (Figure 4C). Additionally, the waterfall plots were utilized to demonstrate the somatic alterations landscapes of the top 20 genes that frequently mutated in GBM samples between low‐ and high‐risk groups, respectively (Figure 4D,E), which illustrated that there is a difference in gene mutation frequency within the two groups.

Immune characteristics of GBM patients in the low‐ and high‐risk groups were obviously different. Moreover, to identify immune cell types that were closely correlated with the prognosis of GBM patients, CIBERSORT algorithm and risk data were integrated and the distribution of 22 immune cell types in 155 patients from low‐ and high‐risk groups was displayed in the bar plot (Figure 4F). In addition, the difference of immune cell proportion between the low‐ and high‐risk groups was illustrated in a box diagram (Figure 4G). NK cells activated (*p* < 0.05), Macrophages M2 (*p* < 0.05), and Mast cells resting (*p* < 0.05) were indicated to be increased obviously in low‐risk group. Based on immune cell type fraction, the ssGSEA scores of immune functions in each sample were calculated (Figure 4H). Kaplan–Meier survival analysis confirmed the correlation between multiple immune cell types/immune function and clinical outcomes (Figure S5).

### 3.5 External validation of the prediction model

**FIGURE 5:**
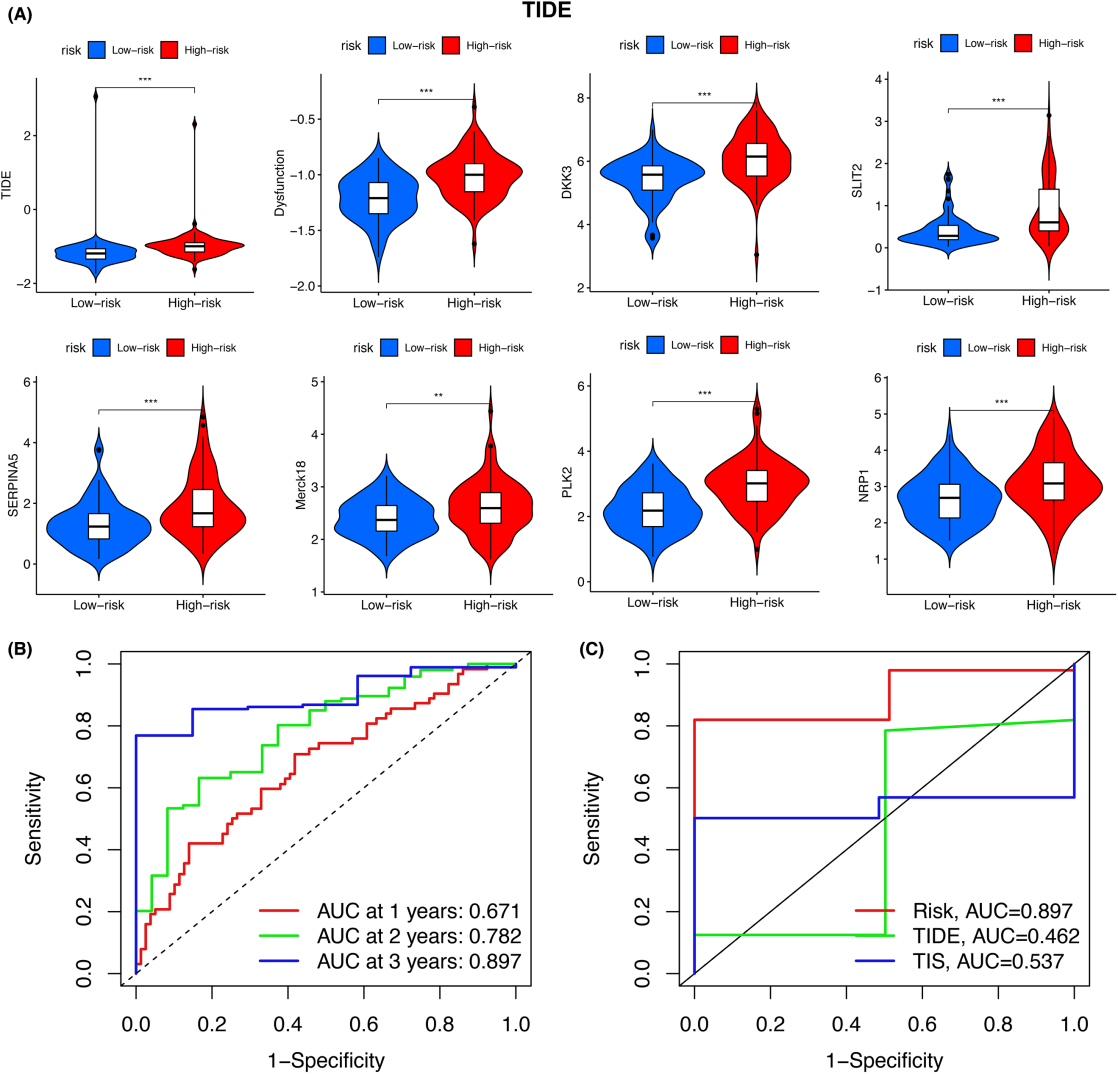
External validation. (A) Violin plots showing the distinction of profiling from Tumor Immune Dysfunction and Exclusion (TIDE) database. The significant difference was identified in biological processes in terms of TIDE (*p* < 0.001), Dysfunction (*p* < 0.001), DKK3 (*p* < 0.001), SLIT2 (*p* < 0.001), SERPINA5 (*p* < 0.001), Merck18 (*p* < 0.01), PLK2 (*p* < 0.001) and NRP1 (*p* < 0.001). (B) Area under the curve (AUC) of receiver operating characteristic (ROC) curve at 1, 2, 3 year was 0.671, 0.782, 0.897, respectively. (C) Area under the curve (AUC) of receiver operating characteristic (ROC) curve of risk score and those from TIDE and T‐cell‐inflamed signature (TIS) was 0.897, 0.462 and 0.537 separately.

To further confirm the reliability of the prediction model, data from TIDE database were retrieved. TIDE evaluated the possible clinical efficacy of immunotherapy in different GBM subgroups. The higher the TIDE score, the worse the Immuno‐checkpoint inhibitors (ICIs) effect and the greater the possibility of immune evasion. Violin plots revealed the significant difference in biological processes in terms of TIDE (*p* < 0.001), Dysfunction (*p* < 0.001), DKK3 (*p* < 0.001), SLIT2 (*p* < 0.001), SERPINA5 (*p* < 0.001), Merck18 (*p* < 0.01), PLK2 (*p* < 0.001) and NRP1 (*p* < 0.001) (Figure 5A). Additionally, the AUCs of TIDE score at 1, 2, 3 year was 0.671, 0.782, 0.897, respectively (Figure 5B). The AUC of risk score, TIDE and TIS was 0.897, 0.462 and 0.537 separately, which indicated that the accuracy of our risk score‐based prognosis prediction model was the best (Figure 5C). TIDE could be utilized to determine two immune evasion mechanisms which induced dysfunction of T cell in cancers with high cytotoxic T lymphocytes (CTL) invasion and preventing T‐cell invasion in cancers with low CTL infiltrates. In melanoma researches, TIDE was identified to be more accurate than other markers, including mutant load and PD‐L1 expression, in the prognosis of first‐line anti‐CTLA4 and/or anti‐PD1 antibody treatment.^16^ Through comparing the predictive accuracy of risk score with TIDE, we identified that risk score exhibited a better predictive capability.

In specific, TIS is referred to as an 18‐gene signature (CCL5, GZMK, CD3D, CD3E, CD2, HLA‐DRA, IL2RG, NKG7, CIITA, CXCR6, LAG3, TAGAP, HLA‐E, CXCL13, IDO1, CXCL10, STAT1, and GZMB), which was related to the response to ICIs in various cancers.^21^, ^22^

### 3.6 Establishment of the regulatory network and identification of targeted inhibitors

**FIGURE 6:**
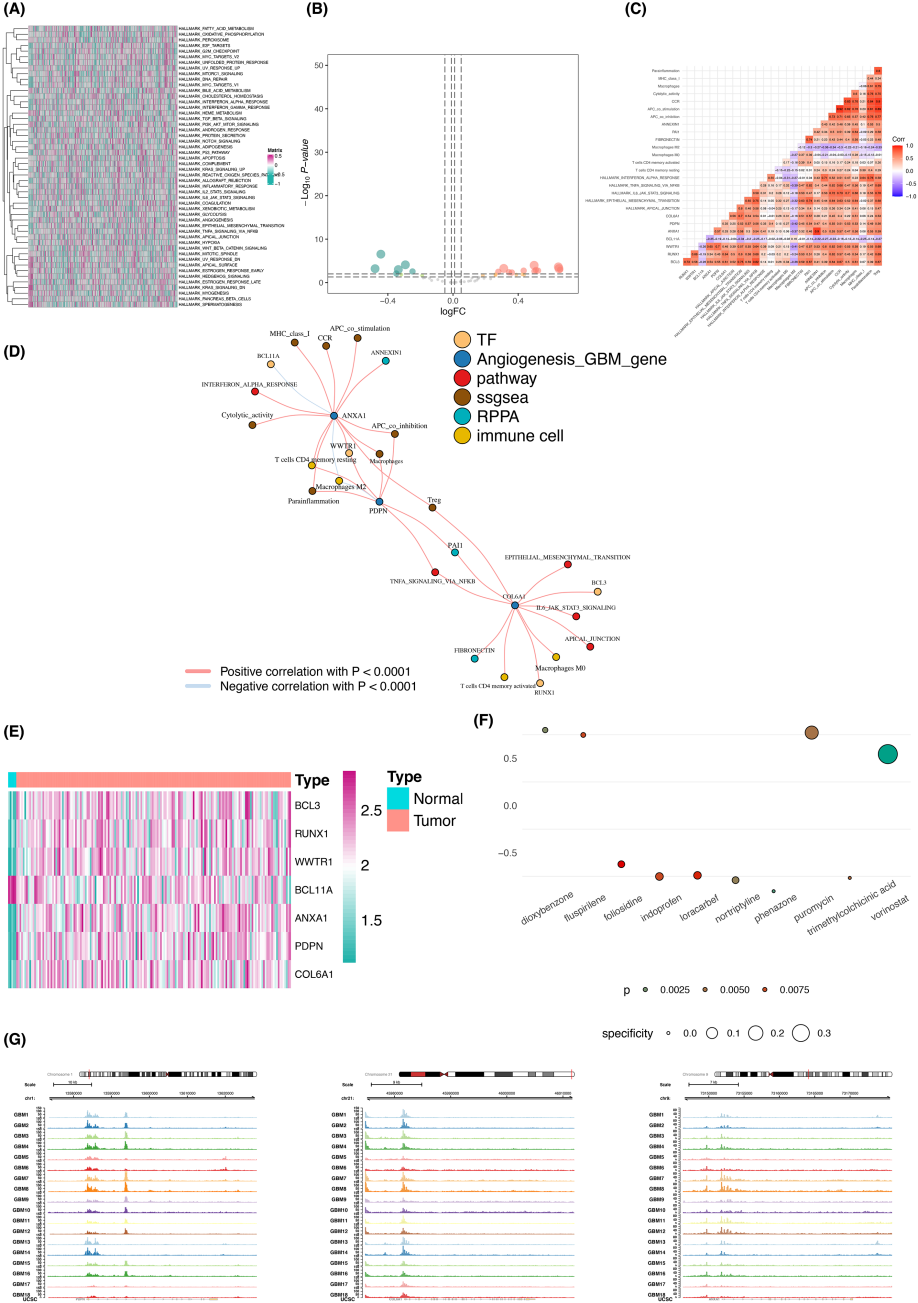
Construction of regulatory network and identification of specific agents. (A) Heatmap plot showing the expression level of 50 hallmark pathways in tissues from GBM patients. (B) Volcano plot displaying hallmark pathways meeting the threshold. (C) heatmap illustrating correlation coefficient of correlation pairs of multi‐omics components. (D) Regulatory network visualizing intercommunication of multi‐omics components. (E) Heatmap exhibiting expression of 3 prognostic differentially expressed angiogenesis‐related genes and 4 differentially expressed transcription factors. (F) Bubble plot displaying top 10 agents with specificity from connectivity map (CMap) database. In these small molecule drugs, phenazone, nortriptyline, foliosidine, trimethylcolchicinic acid, and loracarbef showed closely higher negative correlation. (G) The chromatin accessibility of Annexin A1 (ANXA1), Collagen type VI alpha 1 (COL6A1) and Podoplanin (PDPN).

To unveil the implicit mechanism underlying undesirable clinical outcomes, 50 Hallmark pathways were downloaded and the gene set variation analysis (GSVA) algorithm was adopted. The absolute value of signaling pathways in 173 GBM samples was quantified and displayed in heatmap plot (Figure 6A). Hallmark pathways with significant difference between the neoplastic and normal tissues were illustrated in Figure 6B. We calculated the correlation coefficient of genes and members from multidimensional level and the co‐expression pairs that meet the threshold were identified (Figure 6C). Based on those pairs and their coefficients, 3 PDEARGs (ANXA1, PDPN, and COL6A1), 4 DETFs, 3 RPPA chips, 4 immune cell types, 8 immune functions, and 5 signaling pathways was included in the regulatory network (Figure 6D). We compared the transcriptome data between normal samples and GBM tumor samples, which indicated that ANXA1, PDPN, COL6A1, BCL3, RUNX1, and WWTR1 were upregulated and compared to normal samples, BCL11A was downregulated in GBM tumor samples (Figure 6E). Moreover, to help clinical management and shed light on therapy strategies, data on inhibitors from CMap database was retrieved and the top 10 inhibitors targeting PDEARGs and/or DETFs in the network were found (instances >10, *p* value <0.05, and enrichment score <0). Within these small molecule drugs, phenazone, nortriptyline, foliosidine, trimethylcolchicinic acid, and loracarbef showed closely higher negative correlation, which exhibited a therapeutic potent against GBM (Figure 6F).

We further evaluated the accessible chromatin landscape (or peak calling) representing potential regulatory regions, likely involved in the mechanisms underlying key PDEARG‐driven GBM angiogenesis, using the ATAC‐seq profiles of GBM patients' biopsies from GEO cohort. Over the whole genome across samples, ANXA1, COL6A1 and PDPN were all in the presence of substantial changes in chromatin accessibility widely presenting (Figure 6G).

### 3.7 Association of the expression of PDEARGs with the prognosis of GBM patients

**FIGURE 7:**
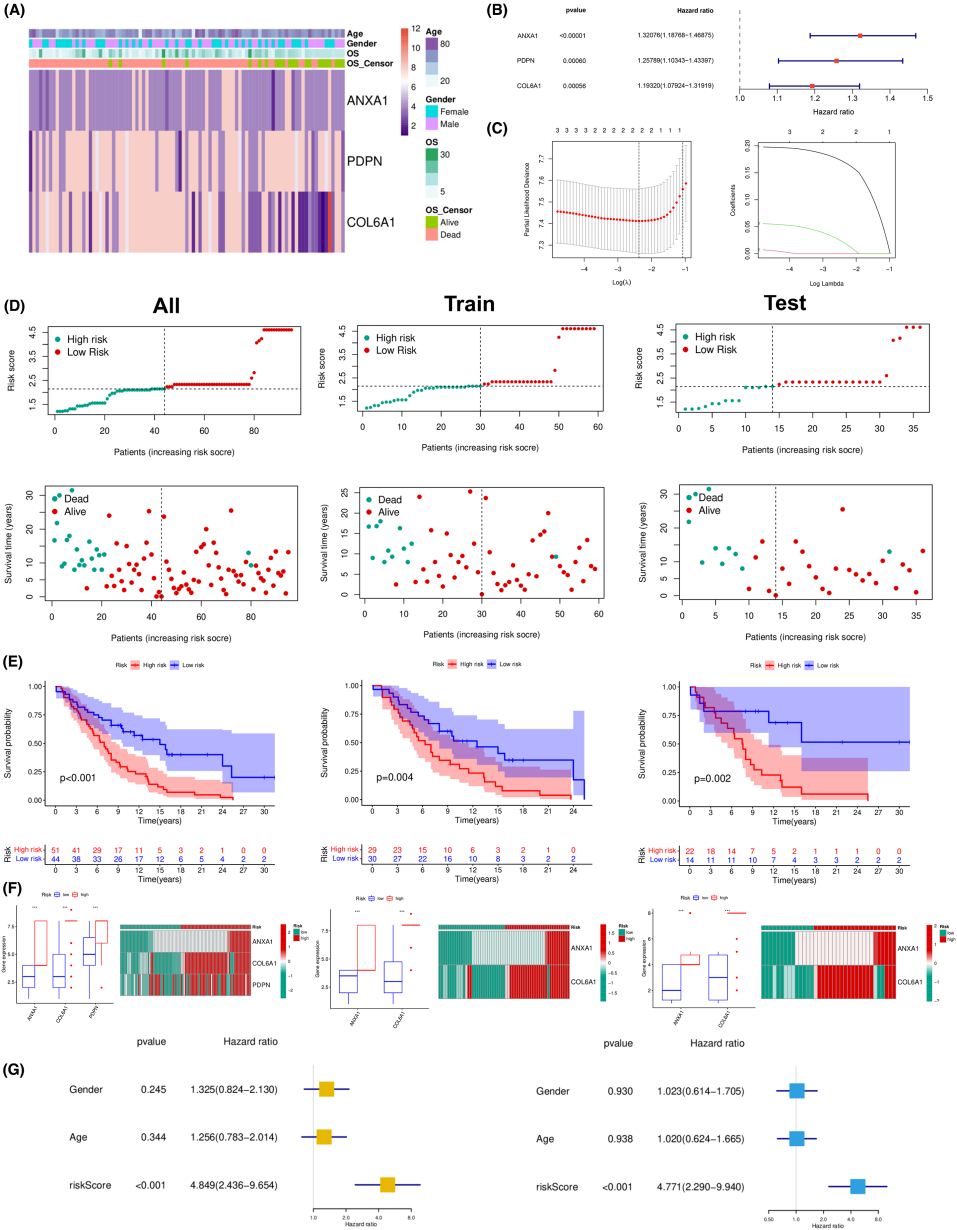
Correlation between PDEARGs and prognosis of glioblastoma patients. (A) Heatmap showing the mRNA expression of 3 prognostic differentially expressed angiogenesis‐related genes. (B) Forest plot displaying hazard ratio of 3 prognostic differentially expressed angiogenesis‐related genes. (C) Lasso plot and lambda plot of the Lasso regression analysis. (D) Risk curve and scatter plot exhibiting risk score and survival status of glioblastoma patients. (E) Kaplan–Meier survival analysis showing the difference of survival probability between low‐ and high‐risk group. (F) Box plot and heatmap plot displaying expression of prognostic differentially expressed angiogenesis‐related genes. (G) Univariate and multivariate Cox regression analysis confirming risk score as an independent prognostic factor.

The mRNA expression of the 3 PDEARGs in GBM patients was shown in Figure 7A. The forest chart demonstrated that these 3 PDEARGs were risk factors (Figure 7B, all *p* < 0.001). We randomly divided the internal group into an internal train group and test group. Subsequently, LASSO regression was employed, and cross‐validation was performed in the internal training group (Figure 7C). The initial signature included three PDEARGs: ANXA1, PDPN, COL6A1. Then, we used multivariate Cox regression to establish a prognostic signature based on these PDEARGs. Eventually, a signature of 2 PDEARGs was selected, and risk score was calculated as follows: risk score = (0.170 * exp of ANXA1) + (0.021 * exp of COL6A1). After calculating the risk score of individual GBM patients, 2.147 was chosen as the cutoff value to distinguish the low‐ and high‐risk groups, helping us to identify the tumor proliferation and the degree of angiogenesis (Figure 7D). Survival analysis showed striking differences between the two groups in train set (*p* = 0.004), test set (*p* = 0.002), and all set (*p* < 0.001) (Figure 7E). Besides, the mRNA expression of ANXA1, PDPN showed a significant difference between the two groups in train and test set, and the 3 PDEARGs expressions were all expressed differentially between the two groups in the whole cohort (Figure 7F, all *p* < 0.001). The risk prediction model could effectively differentiate between the low‐risk group and high‐risk group, and the PDEARG signature was highly stable and effective in different situations.

We conducted univariate and multivariate Cox regression analyses to identify prognosis‐related factors for GBM patients. Forest maps (Figure 7G) indicated that the risk score was independently relevant to the prognosis of GBM patients for univariate Cox regression analysis (HR = 4.849, 95% CI = 2.436–9.654; *p* < 0.001) and multivariate Cox regression analysis (HR = 4.771, 95% CI = 2.290–9.940; *p* < 0.001).

### 3.8 Aggregation and analysis of clinical outcomes from GBM patients

The basic information of 95 GBM patients collected from our center is summarized in Table 1. Among them, 53 cases (55.8%) were male and 42 cases (44.2%) were female; 48 cases (50.1%) under 6 years of age and 47 cases (49.9%) over 6 years of age; The mean HE Score was 4.5, with a 95% confidence interval of 3.923 to 4.666; The mean postoperative OS was 9.287 months, with a 95% confidence interval of 7.916 to 10.660.

**TABLE 1:** Basic clinical characteristics of GBM patients.

Basic characteristics	Classification	Total
*n*	‐	95
Gender (*n* [%])	Male	53 (55.8%)
	Female	42 (44.2%)
Age (*n* [%])	>55	61 (64.2%)
	≤55	34 (35.8&)
HE score	‐	Average value = 4.5 95% CI: 3.923–4.666
OS (month)	‐	Average value = 9.287 95% CI: 7.916–10.660

To explore the correlation between ANXA1, COL6A1 and PDPN at protein level and the prognoses of these GBM patients, we conducted large cohort retrospective analysis with IHC assay. The protein expression levels of ANXA1, COL6A1, and PDPN were negatively correlated to the prognosis of GBM patients (Figure S6A). The K‐M survival analysis proved that GBM patients with higher protein expression of PDEARGs have shorter overall survival time (Figure S6B). The verification results coincided with our previous findings.

In addition, gender, age, Ki‐67 positive rate, HE staining score, and ANXA1, COL6A1, and PDPN staining positivity rates (PC), H‐S, immune‐related risk score (IRS), postoperative survival time (OS, months), and survival status was summarized in Table 2.

**TABLE 2:** Cox regression analysis of risk factors affecting survival time (*n* = 95).

Clinicopathological factors	Univariate analysis			Multivariate analysis		
	HR	95% CI	*p* value	HR	95% CI	*p* value
Age (>55 vs. ≤55)	1.205	0.733–1.981	0.463	1.026	0.593–1.777	0.926
Gender (Male vs. Female)	1.323	0.823–2.162	0.248	1.172	0.655–2.099	0.593
Tumor size (≥3 cm vs. <3 cm)	0.613	0.374–1.006	0.053	0.716	0.414–1.237	0.231
Tumor site						
Frontal sinus (vs. cerebellum).	0.295	0.069–1.262	0.100			
The corpus callosum (vs. cerebellum).	0.503	0.107–2.369	0.385			
The parietal‐occipital lobe (vs. cerebellum).	0.344	0.078–1.513	0.158			
The lateral ventricle (vs. cerebellum).	0.445	0.040–4.989	0.511			
Brainstem‐spinal cord (vs. cerebellum)	0.288	0.040–2.097	0.219			
IDH mutations (mutant vs. wild‐type).	0.597	0.359–0.992	0.047	0.613	0.349–1.067	0.088
p53 mutation (with vs. without).	1.769	0.969–3.227	0.063	1.644	0.856–3.234	0.133
Ki‐67 positivity rate (>25% vs. ≤25%)	0.918	0.569–1.483	0.727	1.272	0.655–2.470	0.477
HE Score (>4.3 vs. ≤4.3)	0.922	0.581–1.463	0.730	0.883	0.474–1.645	0.695
ANXA1						
Positivity rate (>90% vs. ≤90%)	1.514	0.894–2.564	0.123	1.041	0.471–2.300	0.920
Staining score (>110 vs. ≤110)	1.796	1.033–3.123	0.038	1.501	0.725–3.106	0.274
Immune‐related risk scores (>4 vs. ≤4)	1.713	0.945–3.074	0.071	1.258	0.631–2.507	0.514
COL6A1						
Positivity rate (>90%vs. ≤90%)	1.664	1.050–2.638	0.030	1.691	0.860–3.328	0.128
Staining score (>110 vs. ≤110)	1.288	0.785–2.114	0.316	0.965	0.380–2.453	0.940
Immune‐related risk scores (>4 vs. ≤4)	1.252	0.768–2.042	0.368	0.779	0.306–1.978	0.695
PDPN						
Positivity rate (>90% vs. ≤90%)	1.130	0.701–1.821	0.616	1.066	0.581–1.958	0.836
Staining score (>110 vs. ≤110)	0.738	0.349–1.561	0.426	0.883	0.337–2.313	0.800
Immune‐related risk scores (>4 vs. ≤4)	0.950	0.573–1.572	0.841	0.764	0.349–1.672	0.500

### 3.9 Tumor‐related functional states regulated by BRCA subtype‐ specific markers

ANXA1 expression was related to the 14 functional states in nine cancer types (Figure S7A). Then, we compared ANXA1 expression in 23 cancer cell lines among the 14 tumors, in which the GBM cancer cell lines exhibited a high ANXA1 expression. Additionally, based on scRNA‐seq data, it showed significantly positive correlations between ANXA1 and angiogenesis (*R* = 0.27, *p* < 0.01). Moreover, based on the operative specimens from five GBM patients (MGH26, MGH28, MGH29, MGH30, and MGH31), ANXA1 may promote metastasis (*R* = 0.21, *p* < 0.001) and inflammation (*R* = 0.21, *p* < 0.001) in GBM, which may lead to a poor prognosis in GBM patients. Further, it showed significantly strong positive correlations between ANXA1 and metastasis (*R* = 0.36, *p* < 0.01), DNA repair (*R* = 0.31, *p* < 0.01), hypoxia (*R* = 0.30, *p* < 0.01).

COL6A1 expression was associated with the 14 functional states in 15 cancer types (Figure S7B). We next compared COL6A1 expression in 21 cancer cell lines across the 15 cancer types, in which the GBM cancer cell lines exhibited a high COL6A1 expression. Furthermore, it showed significantly strong positive correlations between COL6A1 and inflammation (*R* = 0.24, *p* < 0.001). Furthermore, based on the operative specimens from five GBM patients (MGH26, MGH28, MGH29, MGH30, MGH31), we identified significantly strong positive correlations between COL6A1 and hypoxia (*R* = 0.30, *p* < 0.01).

PDPN expression was correlated with the 14 functional states in 10 cancer types (Figure S7C). We next compared PKIB expression in 15 cancer cell lines across the 10 cancer types. Furthermore, it suggested significantly strong positive correlations between PDPN and metastasis (*R* = 0.38, *p* < 0.001), EMT (*R* = 0.33, *p* < 0.001). Furthermore, based on the operative specimens from five GBM patients (MGH26, MGH28, MGH29, MGH30, and MGH31), we identified significantly strong positive correlations between PDPN and DNA repair (*R* = 0.41, *p* < 0.01), invasion (*R* = 0.33, *p* < 0.01), stemness (*R* = 0.32, *p* < 0.01), metastasis (*R* = 0.33, *p* < 0.05).

### 3.10 Validation of the clinical correlation from the CGGA

To further validate the prognostic value of the identified prognostic differentially expressed angiogenesis‐related genes for GBM, we downloaded and analyzed the sequencing data and clinical information of 389 GBM patients from the CGGA database. Because of missing information, a total of 290 eligible patients were ultimately enrolled in the prognostic analysis. Interestingly, consistent with our previous findings, GBM patients with high ITGA5, COL6A1, SERPINA5, NRP1, PLK2, ANXA1, and PDPN expression had worse survival outcomes, whereas GBM patients with low MAPK1 and HEY1 showed worse survival outcomes (Figure S8).

### 3.11 Initial clustering and cell type annotation

**FIGURE 8:**
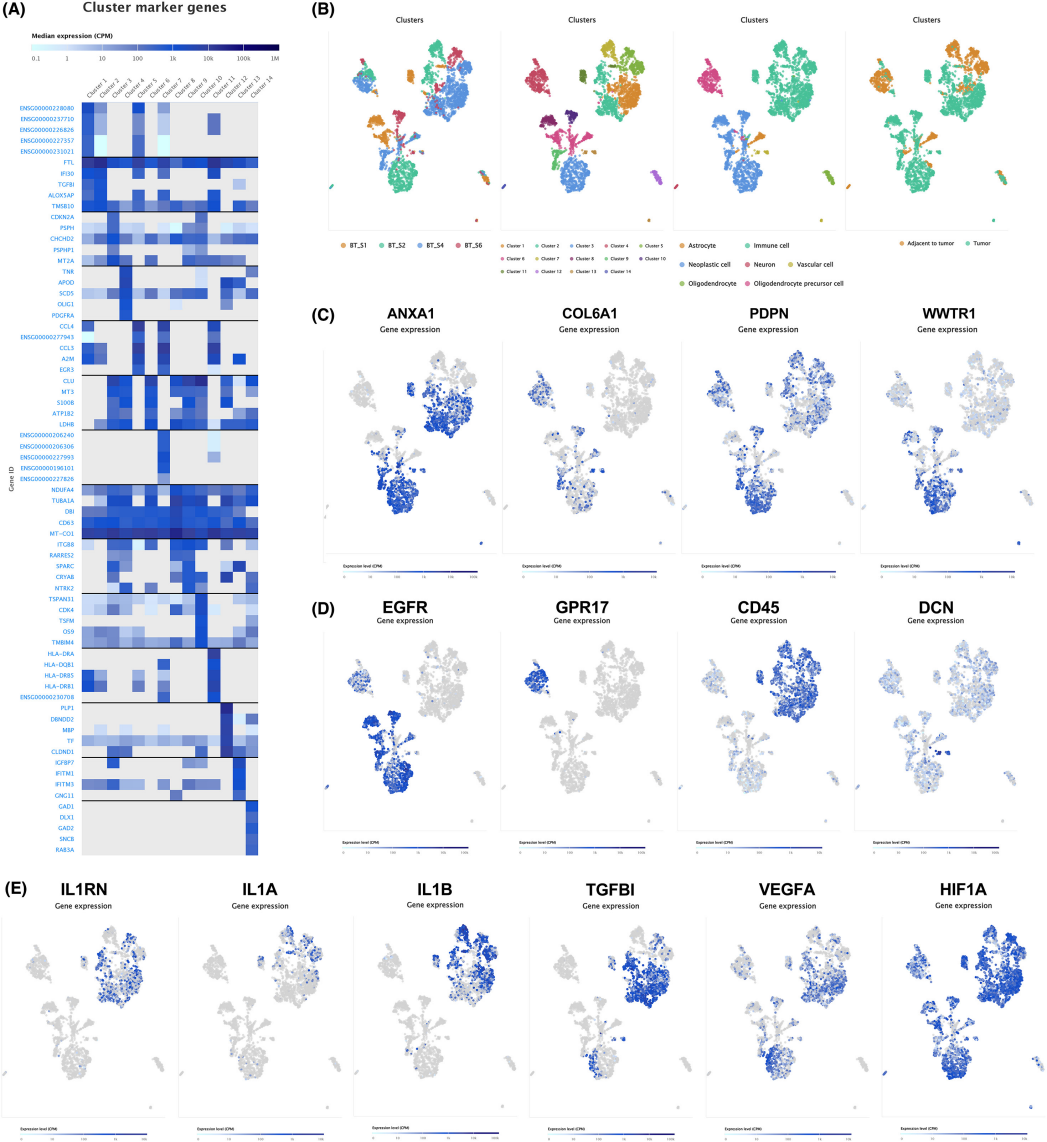
Single‐Cell RNA‐Seq Analysis of glioblastoma Infiltrating Cells. (A) Hierarchical clustering of all single cells based on expression of their gene markers. (B) 2D‐tSNE representation of all single cells included in the study (*n* = 3588). Cell clusters are differentially colored and identified as distinct cell classes. (C) Expression of the 3 prognostic differentially expressed angiogenesis‐related genes and key differentially expressed transcription factors overlaid on the 2D‐tSNE space. (D) Expression of characteristic cell‐type‐specific gene markers overlaid on the 2D‐tSNE space. (E) 2D‐tSNE representation of immune cells from the tumor and periphery for gene markers involved in immune regulation, angiogenesis, and extracellular matrix (ECM) remodeling.

To characterize GBM infiltrating tumor cells, high‐depth scRNA‐seq data on a cohort containing four GBM patients (diagnosed by pathological results, IDH1‐negative) were collected and analyzed, namely BT S1, BT S2, BT S4, and BT S6. Almost all single cells collected from dissociated GBM tumor core attributed to the neoplastic section, instead of other neuronal, glial, vascular, and immune cell subtypes. The cellular identities of the resulting clusters were inferred through identifying significantly over‐expressed genes of the corresponding cell cluster (Figure 8A). Moreover, to verify consistent cell‐type classifications of each cell cluster, we validated the identities of every cluster via combination the dataset and scRNA‐seq data published from normal brain samples.^23^ After QC, dimensional reduction was utilized to construct a feature plot of total 3588 single cells, for visualizing the transcriptomic landscape of every cell. Specifically, the highly over‐dispersion genes (*n* = 500) were selected to generate a cell‐to‐cell dissimilarity matrix. Subsequently, tSNE was conducted on the distance matrix to construct a feature plot of these single cells. Eventually, k‐means clustering was used on the feature plot, and 14 different cell types in distinct clusters were identified (Figure 8B). Importantly, ~94% of these single cells (1029 out of 1091) in malignant cell clusters (neoplastic cluster) were captured from the tumor core. 1029 out of 2343 cells that originated from tumor core were from the malignant cell clusters (~44%). The rest of the cells (*n* = 1182, ~50%) were members of immune cell clusters, with the residual single cells annotated as the oligodendrocyte precursor cell (OPC) cluster (*n* = 50, 2.13%), vascular cell clusters (*n* = 47, 2%), and the oligodendrocyte cluster (*n* = 34, ~1.5%), and neuronal cell cluster (*n* = 1, ~0.05%). It is noteworthy that, the mature astrocyte cluster was the only cluster without any cell found in the malignant tissue. The interaction between these cell types in microenvironment of the tumor may contribute to tumor chemoresistance and progression.

### 3.12 Single‐cell transcriptomic landscape of GBM infiltrating tumor cells

Specifically neoplastic clusters expressed high levels of the 3 PDEARGs (ANXA1, COL6A1, PDPN) and key DETF (WWTR1). Therefore, scRNA‐seq revealed co‐localization of these key biomarkers in malignant cells, and indicated that the interaction between these PDEARGs and DETF may contribute to malignant behavior of GBM cells (Figure 8C). Moreover, EGFR was obviously upregulated in these neoplastic cells, which was overexpressed in 30%–50% of all GBMs.^24^ We also explored gene expression limited to the non‐neoplastic cell clusters and validated the expressing characteristics by using feature plot (Figure 8D). Interestingly, many of the gene markers were routinely used to define mature and differentiated CNS cell types: GPR17 (OPCs), CD45 (myeloid cells), and DCN (vascular cells).^20^

We also identified discrete differences of gene expression between malignant cells and myeloid cells. Therefore, we found more pro‐inflammatory genes were mainly expressed in within periphery tissue and more pro‐angiogenic or anti‐inflammatory gene markers were expressed in the tumor core (Figure 8E). For instance, the pro‐inflammatory gene markers IL1A/B were overexpressed in the peri‐tumor tissue, whereas IL1RN was significantly upregulated in the tumor core, which is an inhibitor of IL1A/B. As a critical anti‐inflammatory factor, IL1RN performs as a negative interactor with IL1R1, which significantly inhibits immune response. Furthermore, a highly expressed marker was TGFBI in the tumor core of immune cells, which was prolonged considered to be originating from malignant cells.^25^ TGFBI can be excited by TGF‐β, which acts as a critical role in the non‐Smad‐mediated TGF‐beta signaling pathway. TGFBI is implicated in growth and dissemination of tumor cells, given that TGFBI could suppress cellular adhesion and keep survival course of tumor cells via DNA damage (chemoresistance), which is also a critical pro‐angiogenic factor (growth or tumor progression). Moreover, GBM infiltrating immune cells may activate angiogenesis by the overexpression of VEGFA, a hypoxia‐induced angiogenic factor (by HIF1A) which facilitates vascular permeability and growth of endothelial cells.

## 4 DISCUSSION

Here, we employed a set of bioinformatics methods to construct an angiogenesis‐related risk prediction model for patients with GBM. Core genes and relevant upstream and downstream components were discovered to establish a network regulating angiogenesis in GBM. Notably, 3 PDEARGs were found to be intensively correlated to WWTR1 (a TF) and hallmark epithelial mesenchymal transition (a signaling process), the binding strength between WWTR1 and 3 PDEARGs was initially confirmed by ATAC‐seq analysis.

Annexin A1 (ANXA1) is known for its role in immune response and inflammation.^26^, ^27^ In triple‐negative breast tumor, ANXA1 is proved to trigger degranulation of mast cells to excrete substances including VEGF and TGF‐β to promote angiogenesis, through interaction with its receptor, ALX/FPR2.^28^, ^29^, ^30^ Nunzia et al. have previously revealed that ANXA1/EVs (extracellular vesicles, contained in exosome secreted by tumor cells) complex is able to activate the transition of epithelial cells and fibroblasts to more aggressive mesenchymal phenotypes.^31^, ^32^, ^33^ In multiple tumors, overexpressed ANXA1 is correlated with epithelial‐mesenchymal transition (EMT),^29^, ^34^ which is consistent with the result of our study. Plenty of studies have confirmed that expression level of ANXA1 protein is related with the invasiveness of various cancer types.^29^, ^35^, ^36^ Based on our work, we confirm a hypothesis that ANXA1 plays an important role in biological process either angiogenesis or EMT in the context of GBM.

Collagen type VI alpha 1 (COL6A1) is a part of a family of collagen and is widely distributed in various tissues including skeletal muscles, skin and blood vessels.^37^ In osteosarcoma (OS), COL6A1 is packed with exosomes and excreted from tumor cells to stimulate fibroblasts to convert into CAFs, which in turn promotes OS metastasis by secreting TGF‐β.^38^ TGF‐β promotes angiogenesis in late stage of tumorigenesis and boosts tumor growth and metastasis through increasing angiogenesis^39^, ^40^ and the present study, we hypothesize that COL6A1 worsens the prognosis of GBM patients by promoting angiogenesis through interacting with multiple components of extracellular matrix (ECM). OWUSU‐ANSAH et al. have demonstrated that increased expression level of COL6A1 is associated with more aggressive and metastatic capabilities of pancreatic tumor cell line, indicating enhanced EMT.^41^ Therefore, COL6A1 proved to be involved in the key process of GBM angiogenesis.

Podoplanin (PDPN) is known to be upregulated in tumor cells, tumor‐associated fibroblasts and inflammatory macrophages.^42^ It has been known as one of the overexpressed gene signatures and is responsible for migration and angiogenesis in GBM tumor samples.^43^, ^44^ Its expression level in high‐grade gliomas was found to worsen the prognosis of patients; thus, in recent years, it has attracted more interest in how it affects tumor growth. It has been revealed that PDPN expression level is upregulated by TGF‐β in other cancer types.^45^, ^46^ The binding of PDPN and its receptor, C‐type lectin‐like receptor 2 (CLEC‐2), specifically distributed on platelet/megakaryocytes in human, activates platelet to release cytokines including TGF‐β,^47^, ^48^ thus facilitating angiogenesis and EMT. Consequently, aforementioned mechanism may inspire the exploration of regulatory significance of PDPN in GBM.

In a word, pro‐angiogenesis and protumor effect of ANXA1, COL6A1 and PDPN are confirmed by previous and the present literature. Nevertheless, finding out how these three key genes interact with one another and whether transcriptional regulators mediating these PDEARGs in GBM angiogenesis turn to be seductive. To our knowledge, few studies have reported the potential impact of ANXA1, COL6A1, and PDPN on the prognosis of GBM patients. Using the risk score, related to degree of proliferation (Ki‐67) and angiogenesis (HE), and IHC to further validate protein expression levels of these genes in our GBM brain tissue samples, we confirmed that ANXA1, COL6A1 and PDPN are not only significantly upregulated in tumor tissues of high‐risk GBM patients, but are closely related to worsened prognosis, indicating their contributions to the pathogenesis of GBM. Furthermore, comprehensive analyses have shown that ANXA1, COL6A1 and PDPN appeared to be promising candidates as prognostic predictors and therapeutic drug targets.

We identified that WWTR1 is closely correlated with the three PDEARGs, which has attracted our interest. WWTR1 is also known as TAZ, which is a transcriptional co‐activator with a PDZ binding motif. YAZ and its paralog the Yes‐associated protein (YAP) are both initially recognized as downstream effectors of the Hippo signaling pathways. In the context of various human tumors, TAZ/TAP have been activated and translocated into the nucleus to regulate target genes expression, enhancing malignant cell proliferation, maintaining a pro‐neoplasm microenvironment and promoting cancer stemness.^49^ Z. Wang et al. have revealed that VEGFC derived from GBM cells facilitates the viability, migration, and tubulation of vessel endothelial cells by suppressing the Hippo signaling pathways to stimulate WWTR1, which confirmed the pro‐angiogenesis significance of WWTR1 in GBM.^50^

The regulatory interaction between WWTR1 and 3 PDEARGs was initially confirmed by ATAC‐seq analysis in the present study. Furthermore, using scRNA‐seq data of 3588 cells in a cohort of 4 GBM patients, single infiltrating tumor cells within peripheral tumor core of GBM were clustered and characterized. Despite the fact that significant heterogeneity exists across neoplastic cells, we identified that infiltrating tumor cells share a consistent gene expression pattern between patients, indicating a common infiltration mechanism. As expected, we found that the value of 3 PDEARGs (ANXA1, COL6A1, and PDPN) and key DETF (WWTR1) were co‐expressed significantly higher in the tumor core than in the peripheral of GBM tissue, specifically in the neoplastic cells. Additionally, we identified that GBM infiltrating immune cells play an important role in promoting tumor cells' growth, survival, and dissemination through inhibition of inflammation, activation of angiogenesis and remodeling of ECM.

Here, we applied function enrichment analysis in search of underlying downstream biological process related to PDEARGs. Among the hallmark events, epithelial mesenchymal transition (EMT) was highly correlated with PDEARGs. EMT enables solid tumor cells to acquire the potential to evade from primary tumor and disseminate through the body by equipping the cells with capabilities to undergo morphological transitions such as the loss of cell–cell adhesion, the loss of cell polarity and the gain of mesenchymal markers.^51^, ^52^, ^53^ Fantozzi et al. have demonstrated that EMT promotes the expression level of genes involved in angiogenesis, such as VEGFA,^54^ which led to our proposal that, the most PDEARGs‐correlated downstream pathway, EMT, enhances angiogenesis in GBM. Strikingly, it is universally acknowledged that among all the inducers to activate EMT, TGF‐β is the most potent.^55^, ^56^

Up to now, the hypothesis chain regarding the regulatory network in our study has been completed, that is, transcriptional regulator WWTR1 up‐regulates the expression level of 3 PDEARGs, which may promote the excretion of TGF‐β, finally resulting in EMT‐mediated angiogenesis in GBM.

Additionally, on the immune cell level, we have found that regulatory T cell (Treg) has the strongest correlation coefficient with the three PDEARGs, indicating that upregulation of PDEARGs may promote the infiltration of Treg, mediating angiogenesis and undesirable outcomes in GBM patients. Abundant studies have confirmed that Treg enhances the establishment of VEGFA‐rich cancer microenvironment and promotes angiogenesis in ovarian cancer,^57^, ^58^ which supports our hypothesis.

Among candidate agents with high specificity identified in this study, Nortriptyline is a potent autophagy inhibitor and has been repurposed as a medication to treat brain cancers such as GBM owing to its ability to permeate brain–blood barrier (BBB) and tumor cells toxicity.^59^ HDAC have been found to be responsible for HIF ‐α induced angiogenesis.^60^ Given the fact that Vorinostat is a histone deacetylases (HDAC) inhibitor, it may block angiogenesis by indirectly inhibiting VEGF and hypoxia inducible factor (HIF) ‐α under hypoxic conditions. Previous study has demonstrated that Vorinostat impairs vasculogenic mimicry, an alternative vascularization mechanism, to interfere with tube formation in GBM.^61^

## 5 CONCLUSIONS

In the present study, we have constructed a regulatory network based on angiogenesis‐related gene signature and identified a signaling pathway, WWTR1 (TAZ)‐ANXA1, COL6A1 and PDPN‐EMT, which may provide new therapeutic strategies by utilizing the signaling axis in tumor angiogenesis we proposed for GBM patients.

## AUTHOR CONTRIBUTIONS

**Zhiping Wan:** Conceptualization (lead). **Xiaokun Zuo:** Conceptualization (equal); formal analysis (equal); methodology (equal). **Siqiao Wang:** Formal analysis (equal); investigation (equal); writing – review and editing (equal). **Lei Zhou:** Methodology (equal). **Xiaojing Wen:** Data curation (equal); investigation (equal). **Ying Yao:** Investigation (equal); methodology (equal). **Jiefang Song:** Data curation (equal). **Juan Gu:** Resources (equal); software (equal); visualization (equal). **Zhimin Wang:** Data curation (equal); funding acquisition (equal); investigation (equal). **Ran Liu:** Investigation (equal); writing – original draft (equal); writing – review and editing (equal). **Chun Luo:** Data curation (equal); funding acquisition (equal).

## FUNDING INFORMATION

This study was funded by Natural Science Foundation of China (grant No.82171832), Shanghai science and Technology Innovation Fund (grant No.20Z11900105).

## CONFLICT OF INTEREST STATEMENT

The authors have no conflict of interest.

## ETHICS STATEMENT

We state that our study was granted by the Ethics Committee of Tongji Hospital affiliated to Tongji University (No. SBKT‐2022‐053), and we conducted the research in strict accordance with its regulations.

## Supporting information


Data S1.

## Data Availability

Data sharing is not applicable to this article as no new data were created or analyzed in this study.

## References

[1] Gimple RC , Bhargava S , Dixit D , Rich JN . Glioblastoma stem cells: lessons from the tumor hierarchy in a lethal cancer. Genes Dev. 2019;33(11–12):591‐609. 31160393 10.1101/gad.324301.119 PMC6546059

[2] Lee JH , Lee JE , Kahng JY , et al. Human glioblastoma arises from subventricular zone cells with low‐level driver mutations. Nature. 2018;560(7717):243‐247. 30069053 10.1038/s41586-018-0389-3

[3] Ostrom QT , Gittleman H , Fulop J , et al. CBTRUS statistical report: primary brain and central nervous system tumors diagnosed in the United States in 2008‐2012. Neuro Oncol. 2015;17(Suppl 4):iv1‐iv62. 26511214 10.1093/neuonc/nov189 PMC4623240

[4] Poon MTC , Sudlow CLM , Figueroa JD , Brennan PM . Longer‐term (≥ 2 years) survival in patients with glioblastoma in population‐based studies pre‐ and post‐2005: a systematic review and meta‐analysis. Sci Rep. 2020;10(1):11622. 32669604 10.1038/s41598-020-68011-4 PMC7363854

[5] Stupp R , Hegi ME , Mason WP , et al. Effects of radiotherapy with concomitant and adjuvant temozolomide versus radiotherapy alone on survival in glioblastoma in a randomised phase III study: 5‐year analysis of the EORTC‐NCIC trial. Lancet Oncol. 2009;10(5):459‐466. 19269895 10.1016/S1470-2045(09)70025-7

[6] Sanai N , Berger MS . Glioma extent of resection and its impact on patient outcome. Neurosurgery. 2008;62(4):753‐764. 18496181 10.1227/01.neu.0000318159.21731.cf

[7] Ahir BK , Engelhard HH , Lakka SS . Tumor development and angiogenesis in adult brain tumor: glioblastoma. Mol Neurobiol. 2020;57(5):2461‐2478. 32152825 10.1007/s12035-020-01892-8 PMC7170819

[8] Gerstner ER , Batchelor TT . Antiangiogenic therapy for glioblastoma. Cancer J. 2012;18(1):45‐50. 22290257 10.1097/PPO.0b013e3182431c6f PMC3269655

[9] Touat M , Idbaih A , Sanson M , Ligon KL . Glioblastoma targeted therapy: updated approaches from recent biological insights. Ann Oncol. 2017;28(7):1457‐1472. 28863449 10.1093/annonc/mdx106 PMC5834086

[10] Zheng R , Wan C , Mei S , et al. Cistrome data browser: expanded datasets and new tools for gene regulatory analysis. Nucleic Acids Res. 2019;47(D1):D729‐D735. 30462313 10.1093/nar/gky1094 PMC6324081

[11] Bhattacharya S , Andorf S , Gomes L , et al. ImmPort: disseminating data to the public for the future of immunology. Immunol Res. 2014;58(2–3):234‐239. 24791905 10.1007/s12026-014-8516-1

[12] Engebretsen S , Bohlin J . Statistical predictions with glmnet. Clin Epigenetics. 2019;11(1):123. 31443682 10.1186/s13148-019-0730-1 PMC6708235

[13] Subramanian A , Kuehn H , Gould J , Tamayo P , Mesirov JP . GSEA‐P: a desktop application for Gene Set Enrichment Analysis. Bioinformatics. 2007;23(23):3251‐3253. 17644558 10.1093/bioinformatics/btm369

[14] Newman AM , Steen CB , Liu CL , et al. Determining cell type abundance and expression from bulk tissues with digital cytometry. Nat Biotechnol. 2019;37(7):773‐782. 31061481 10.1038/s41587-019-0114-2 PMC6610714

[15] Hanzelmann S , Castelo R , Guinney J . GSVA: gene set variation analysis for microarray and RNA‐seq data. BMC Bioinformatics. 2013;14:7. 23323831 10.1186/1471-2105-14-7 PMC3618321

[16] Jiang P , Gu S , Pan D , et al. Signatures of T cell dysfunction and exclusion predict cancer immunotherapy response. Nat Med. 2018;24(10):1550‐1558. 30127393 10.1038/s41591-018-0136-1 PMC6487502

[17] Lamb J , Crawford ED , Peck D , et al. The connectivity map: using gene‐expression signatures to connect small molecules, genes, and disease. Science. 2006;313(5795):1929‐1935. 17008526 10.1126/science.1132939

[18] Buenrostro JD , Wu B , Chang HY , Greenleaf WJ . ATAC‐seq: a method for assaying chromatin accessibility genome‐wide. Curr Protoc Mol Biol. 2015;109:21.29.1‐21.29.9. 10.1002/0471142727.mb2129s109 PMC4374986 25559105

[19] Hahne F , Ivanek R . Visualizing genomic data using gviz and bioconductor. Methods Mol Biol. 2016;1418:335‐351. 27008022 10.1007/978-1-4939-3578-9_16

[20] Darmanis S , Sloan SA , Croote D , et al. Single‐cell RNA‐seq analysis of infiltrating neoplastic cells at the migrating front of human glioblastoma. Cell Rep. 2017;21(5):1399‐1410. 29091775 10.1016/j.celrep.2017.10.030 PMC5810554

[21] Ayers M , Lunceford J , Nebozhyn M , et al. IFN‐gamma‐related mRNA profile predicts clinical response to PD‐1 blockade. J Clin Invest. 2017;127(8):2930‐2940. 28650338 10.1172/JCI91190 PMC5531419

[22] Bertucci F , Boudin L , Finetti P , et al. Immune landscape of inflammatory breast cancer suggests vulnerability to immune checkpoint inhibitors. Onco Targets Ther. 2021;10(1):1929724. 10.1080/2162402X.2021.1929724 PMC8158040 34104544

[23] Darmanis S , Sloan SA , Zhang Y , et al. A survey of human brain transcriptome diversity at the single cell level. Proc Natl Acad Sci U S A. 2015;112(23):7285‐7290. 26060301 10.1073/pnas.1507125112 PMC4466750

[24] Libermann TA , Nusbaum HR , Razon N , et al. Amplification, enhanced expression and possible rearrangement of EGF receptor gene in primary human brain tumours of glial origin. Nature. 1985;313(5998):144‐147. 2981413 10.1038/313144a0

[25] Han B , Cai H , Chen Y , et al. The role of TGFBI (betaig‐H3) in gastrointestinal tract tumorigenesis. Mol Cancer. 2015;14:64. 25889002 10.1186/s12943-015-0335-z PMC4435624

[26] Dalli J , Norling LV , Renshaw D , Cooper D , Leung KY , Perretti M . Annexin 1 mediates the rapid anti‐inflammatory effects of neutrophil‐derived microparticles. Blood. 2008;112(6):2512‐2519. 18594025 10.1182/blood-2008-02-140533

[27] Zhang Z , Huang L , Zhao W , Rigas B . Annexin 1 induced by anti‐inflammatory drugs binds to NF‐kappaB and inhibits its activation: anticancer effects in vitro and in vivo. Cancer Res. 2010;70(6):2379‐2388. 20215502 10.1158/0008-5472.CAN-09-4204 PMC2953961

[28] Feoktistov I , Ryzhov S , Goldstein AE , Biaggioni I . Mast cell‐mediated stimulation of angiogenesis: cooperative interaction between A2B and A3 adenosine receptors. Circ Res. 2003;92(5):485‐492. 12600879 10.1161/01.RES.0000061572.10929.2D

[29] Okano M , Oshi M , Butash AL , et al. Triple‐negative breast cancer with high levels of annexin A1 expression is associated with mast cell infiltration, inflammation, and angiogenesis. Int J Mol Sci. 2019;20(17):4197. 31461932 10.3390/ijms20174197 PMC6747082

[30] Sinniah A , Yazid S , Perretti M , Solito E , Flower RJ . The role of the annexin‐A1/FPR2 system in the regulation of mast cell degranulation provoked by compound 48/80 and in the inhibitory action of nedocromil. Int Immunopharmacol. 2016;32:87‐95. 26803520 10.1016/j.intimp.2016.01.003 PMC4760273

[31] Bu L , Baba H , Yoshida N , et al. Biological heterogeneity and versatility of cancer‐associated fibroblasts in the tumor microenvironment. Oncogene. 2019;38(25):4887‐4901. 30816343 10.1038/s41388-019-0765-y

[32] Gao L , Zhang W , Zhong W‑Q , et al. Tumor associated macrophages induce epithelial to mesenchymal transition via the EGFR/ERK1/2 pathway in head and neck squamous cell carcinoma. Oncol Rep. 2018;40(5):2558‐2572. 30132555 10.3892/or.2018.6657 PMC6151899

[33] Pessolano E , Belvedere R , Bizzarro V , et al. Annexin A1 may induce pancreatic cancer progression as a key player of extracellular vesicles effects as evidenced in the in vitro MIA PaCa‐2 model system. Int J Mol Sci. 2018;19(12):3878. 30518142 10.3390/ijms19123878 PMC6321029

[34] Oshi M , Tokumaru Y , Mukhopadhyay S , et al. Annexin A1 expression is associated with epithelial‐mesenchymal transition (EMT), cell proliferation, prognosis, and drug response in pancreatic cancer. Cell. 2021;10(3):653. 10.3390/cells10030653 PMC8000658 33804148

[35] Novizio N , Belvedere R , Pessolano E , et al. ANXA1 contained in EVs regulates macrophage polarization in tumor microenvironment and promotes pancreatic cancer progression and metastasis. Int J Mol Sci. 2021;22(20):11018. 34681678 10.3390/ijms222011018 PMC8538745

[36] Rubinstein MR , Baik JE , Lagana SM , et al. Fusobacterium nucleatum promotes colorectal cancer by inducing Wnt/β‐catenin modulator annexin A1. EMBO Rep. 2019;20(4):e47638. 30833345 10.15252/embr.201847638 PMC6446206

[37] Cescon M , Gattazzo F , Chen P , Bonaldo P . Collagen VI at a glance. J Cell Sci. 2015;128(19):3525‐3531. 26377767 10.1242/jcs.169748

[38] Zhang Y , Liu Z , Yang X , et al. H3K27 acetylation activated‐COL6A1 promotes osteosarcoma lung metastasis by repressing STAT1 and activating pulmonary cancer‐associated fibroblasts. Theranostics. 2021;11(3):1473‐1492. 33391546 10.7150/thno.51245 PMC7738898

[39] Hao Y , Baker D , Ten Dijke P . TGF‐β‐mediated epithelial‐mesenchymal transition and cancer metastasis. Int J Mol Sci. 2019;20(11):2767. 31195692 10.3390/ijms20112767 PMC6600375

[40] Muppala S , Xiao R , Krukovets I , et al. Thrombospondin‐4 mediates TGF‐β‐induced angiogenesis. Oncogene. 2017;36(36):5189‐5198. 28481870 10.1038/onc.2017.140 PMC5589494

[41] Owusu‐Ansah KG , Song G , Chen R , et al. COL6A1 promotes metastasis and predicts poor prognosis in patients with pancreatic cancer. Int J Oncol. 2019;55(2):391‐404. 31268154 10.3892/ijo.2019.4825 PMC6615918

[42] Krishnan H , Rayes J , Miyashita T , et al. Podoplanin: an emerging cancer biomarker and therapeutic target. Cancer Sci. 2018;109(5):1292‐1299. 29575529 10.1111/cas.13580 PMC5980289

[43] Grau SJ , Trillsch F , Tonn JC , et al. Podoplanin increases migration and angiogenesis in malignant glioma. Int J Clin Exp Pathol. 2015;8(7):8663‐8670. 26339454 PMC4555782

[44] Lu CH , Wei ST , Liu JJ , et al. Recognition of a novel gene signature for human glioblastoma. Int J Mol Sci. 2022;23(8):4157. 35456975 10.3390/ijms23084157 PMC9029857

[45] Ohta M , Abe A , Ohno F , et al. Positive and negative regulation of podoplanin expression by TGF‐β and histone deacetylase inhibitors in oral and pharyngeal squamous cell carcinoma cell lines. Oral Oncol. 2013;49(1):20‐26. 22840788 10.1016/j.oraloncology.2012.06.017

[46] Wu Y , Liu Q , Yan X , et al. Podoplanin‐mediated TGF‐β‐induced epithelial‐mesenchymal transition and its correlation with bHLH transcription factor DEC in TE‐11 cells. Int J Oncol. 2016;48(6):2310‐2320. 27035755 10.3892/ijo.2016.3445 PMC4863730

[47] Suzuki‐Inoue K . Platelets and cancer‐associated thrombosis: focusing on the platelet activation receptor CLEC‐2 and podoplanin. Blood. 2019;134(22):1912‐1918. 31778548 10.1182/blood.2019001388

[48] Suzuki‐Inoue K , Tsukiji N . Platelet CLEC‐2 and lung development. Res Pract Thromb Haemost. 2020;4(4):481‐490. 32548549 10.1002/rth2.12338 PMC7292670

[49] Castellan M , Guarnieri A , Fujimura A , et al. Single‐cell analyses reveal YAP/TAZ as regulators of stemness and cell plasticity in glioblastoma. Nat Cancer. 2021;2(2):174‐188. 33644767 10.1038/s43018-020-00150-z PMC7116831

[50] Wang Z , Yuan Y , Ji X , et al. The hippo‐TAZ axis mediates vascular endothelial growth factor C in glioblastoma‐derived exosomes to promote angiogenesis. Cancer Lett. 2021;513:1‐13. 34010715 10.1016/j.canlet.2021.05.002

[51] Hanahan D , Weinberg RA . Hallmarks of cancer: the next generation. Cell. 2011;144(5):646‐674. 21376230 10.1016/j.cell.2011.02.013

[52] Nguyen DX , Bos PD , Massagué J . Metastasis: from dissemination to organ‐specific colonization. Nat Rev Cancer. 2009;9(4):274‐284. 19308067 10.1038/nrc2622

[53] Polyak K , Weinberg RA . Transitions between epithelial and mesenchymal states: acquisition of malignant and stem cell traits. Nat Rev Cancer. 2009;9(4):265‐273. 19262571 10.1038/nrc2620

[54] Fantozzi A , Gruber DC , Pisarsky L , et al. VEGF‐mediated angiogenesis links EMT‐induced cancer stemness to tumor initiation. Cancer Res. 2014;74(5):1566‐1575. 24413534 10.1158/0008-5472.CAN-13-1641

[55] Peng D , Fu M , Wang M , Wei Y , Wei X . Targeting TGF‐β signal transduction for fibrosis and cancer therapy. Mol Cancer. 2022;21(1):104. 35461253 10.1186/s12943-022-01569-x PMC9033932

[56] Xu J , Lamouille S , Derynck R . TGF‐beta‐induced epithelial to mesenchymal transition. Cell Res. 2009;19(2):156‐172. 19153598 10.1038/cr.2009.5 PMC4720263

[57] Facciabene A , Peng X , Hagemann IS , et al. Tumour hypoxia promotes tolerance and angiogenesis via CCL28 and T(reg) cells. Nature. 2011;475(7355):226‐230. 21753853 10.1038/nature10169

[58] Wright SE , Rewers‐Felkins KA , Quinlin IS , et al. Cytotoxic T‐lymphocyte immunotherapy for ovarian cancer: a pilot study. J Immunother. 2012;35(2):196‐204. 22306908 10.1097/CJI.0b013e318243f213 PMC3276404

[59] Petrosyan E , Fares J , Cordero A , et al. Repurposing autophagy regulators in brain tumors. Int J Cancer. 2022;151(2):167‐180. 35179776 10.1002/ijc.33965 PMC9133056

[60] Kim MS , Kwon HJ , Lee YM , et al. Histone deacetylases induce angiogenesis by negative regulation of tumor suppressor genes. Nat Med. 2001;7(4):437‐443. 11283670 10.1038/86507

[61] Pastorino O , Gentile MT , Mancini A , et al. Histone deacetylase inhibitors impair vasculogenic mimicry from glioblastoma cells. Cancers (Basel). 2019;11(6):747. 31146471 10.3390/cancers11060747 PMC6627137

