# Supplementary figures and images for: Identification of angiogenesis‐related genes signature for predicting survival and its regulatory network in glioblastoma
Source: Cancer Med. 2023 Jul 11;12(16):17445–67. doi: 10.1002/cam4.6316 (PMC10501277; doi:10.1002/cam4.6316)

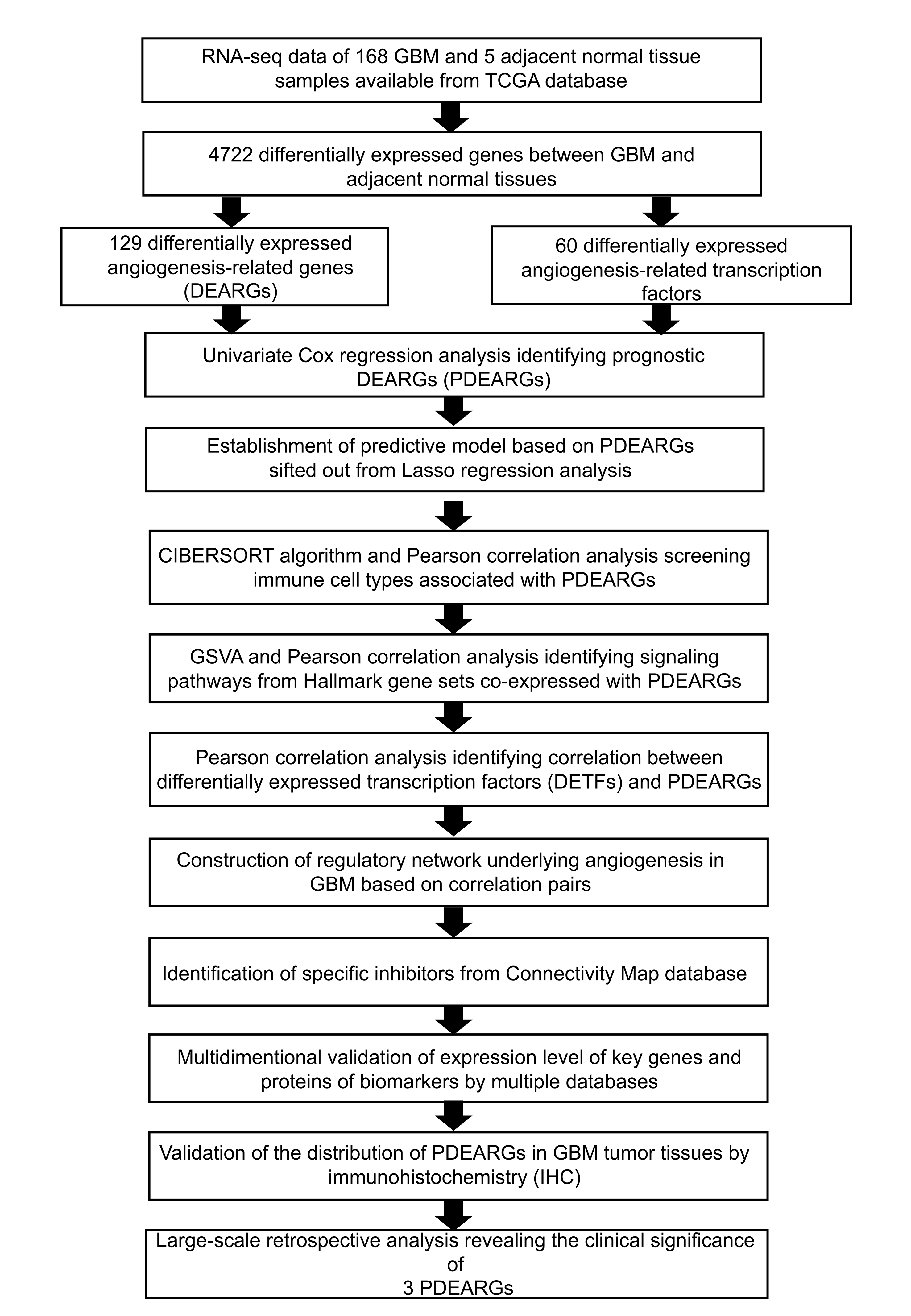

Supplement: Supplementary file 1 — Data S1. [file CAM4-12-17445-s001.zip › cam46316-sup-0001-FigureS1.tif]

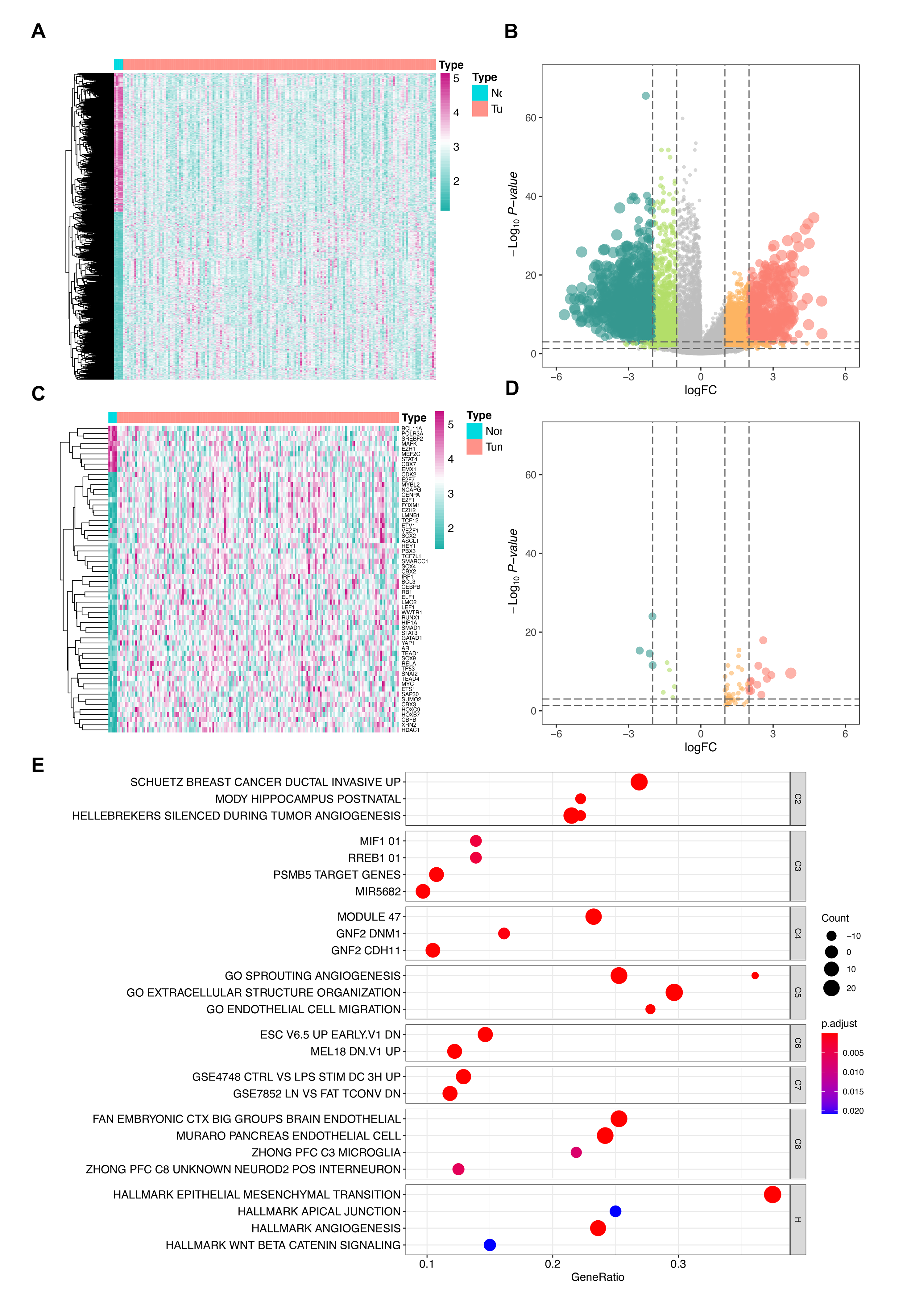

Supplement: Supplementary file 1 — Data S1. [file CAM4-12-17445-s001.zip › cam46316-sup-0002-FigureS2.tif]

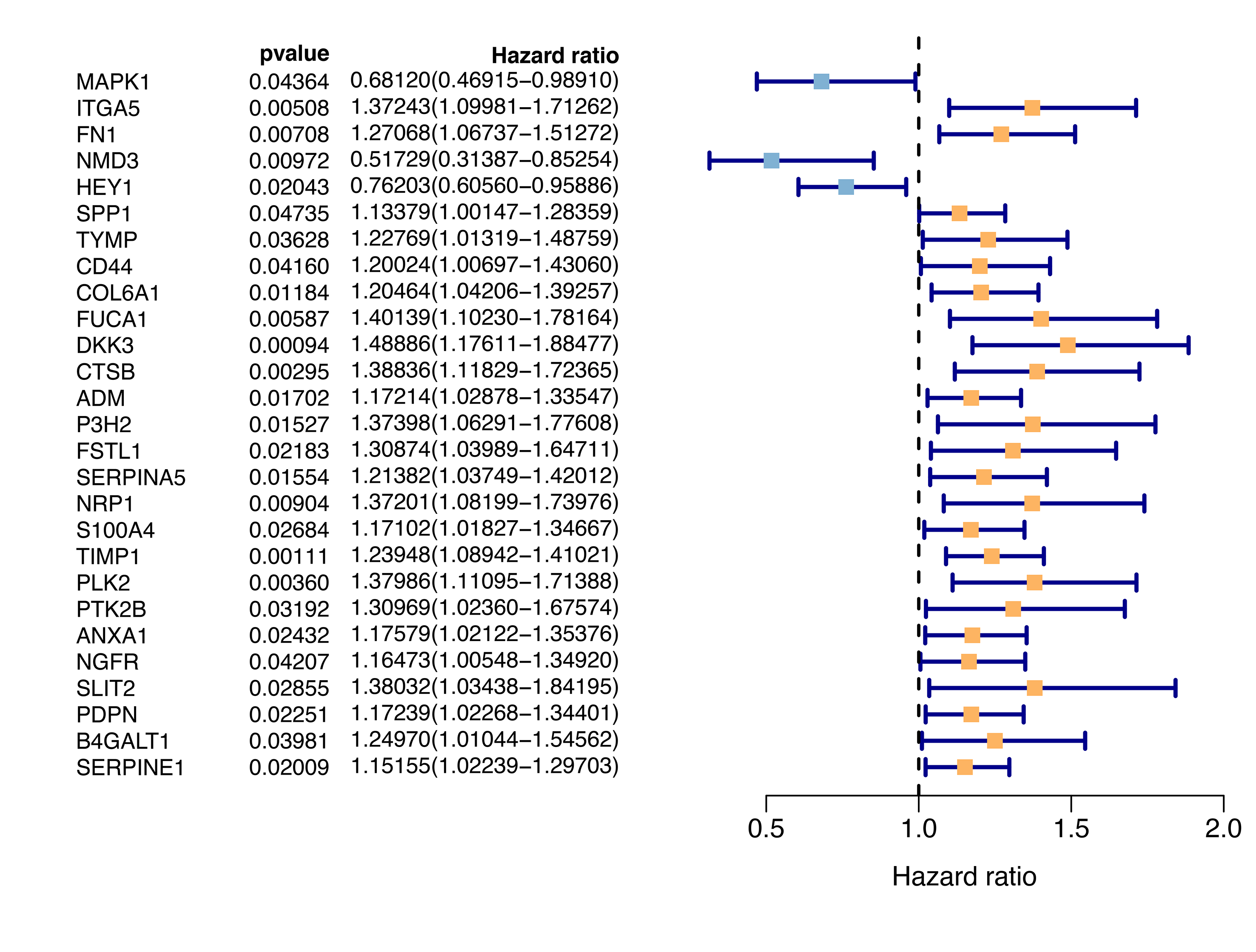

Supplement: Supplementary file 1 — Data S1. [file CAM4-12-17445-s001.zip › cam46316-sup-0003-FigureS3.tif]

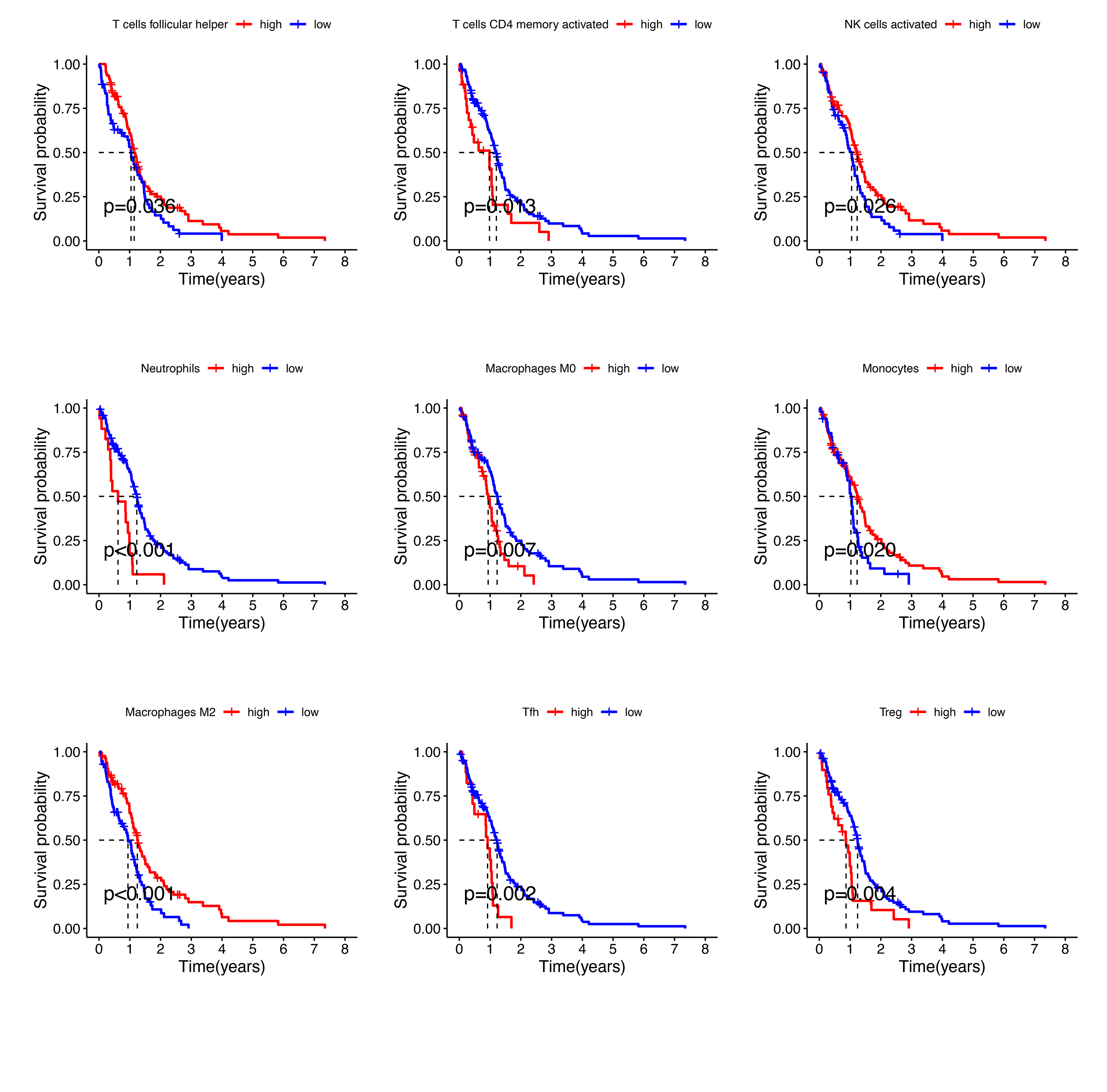

Supplement: Supplementary file 1 — Data S1. [file CAM4-12-17445-s001.zip › cam46316-sup-0004-FigureS4.tif]

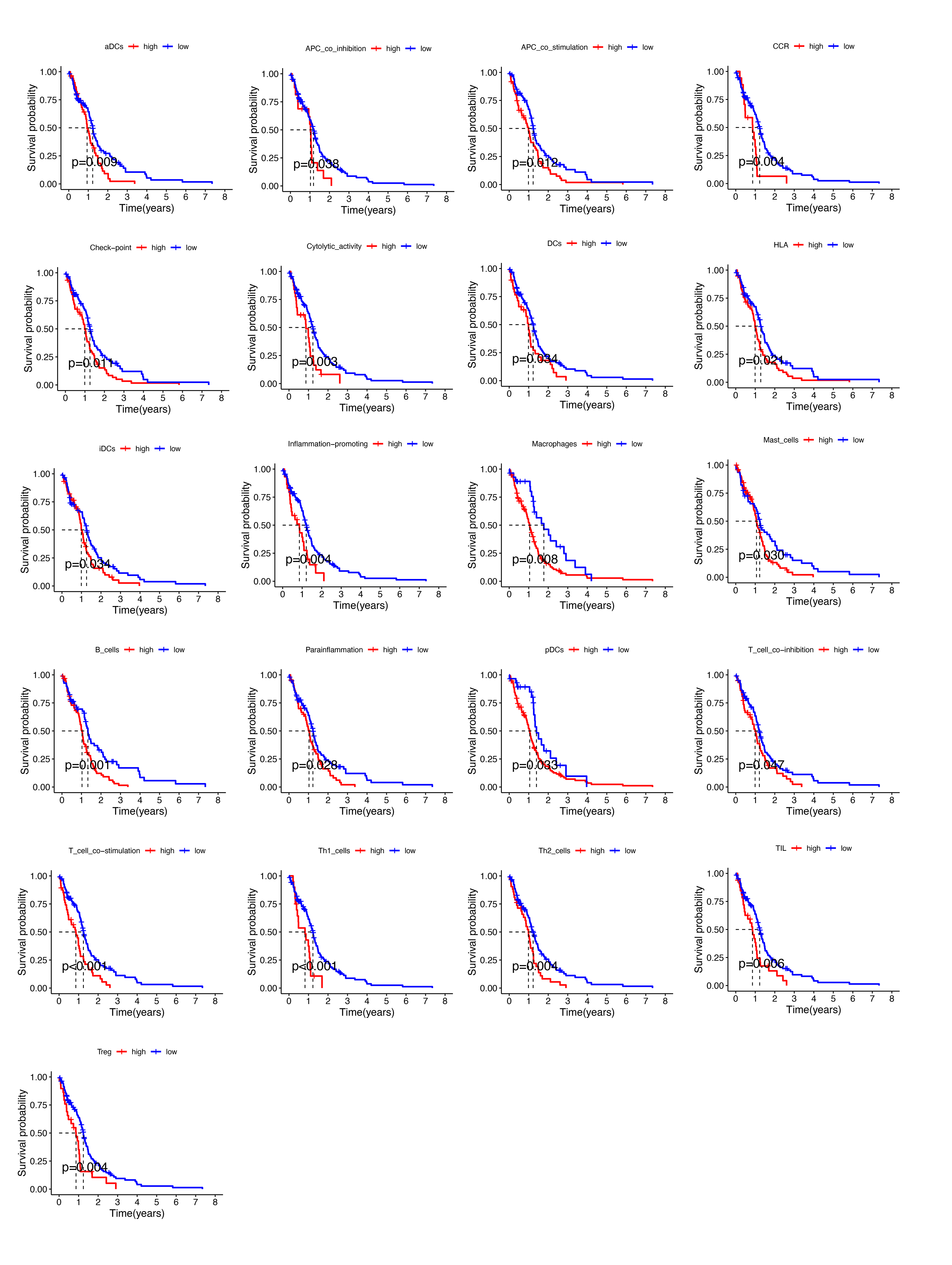

Supplement: Supplementary file 1 — Data S1. [file CAM4-12-17445-s001.zip › cam46316-sup-0005-FigureS5.tif]

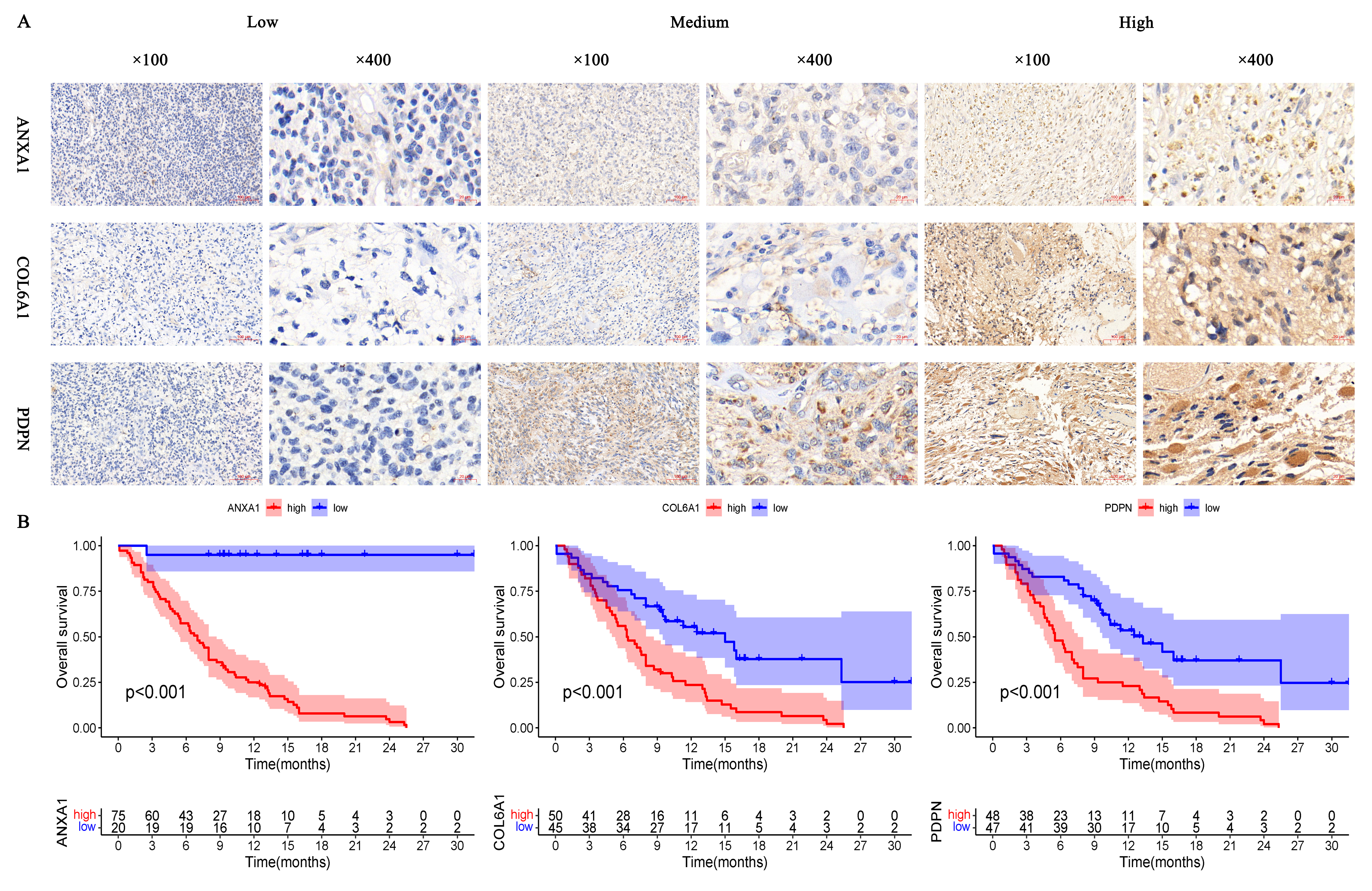

Supplement: Supplementary file 1 — Data S1. [file CAM4-12-17445-s001.zip › cam46316-sup-0006-FigureS6.tif]

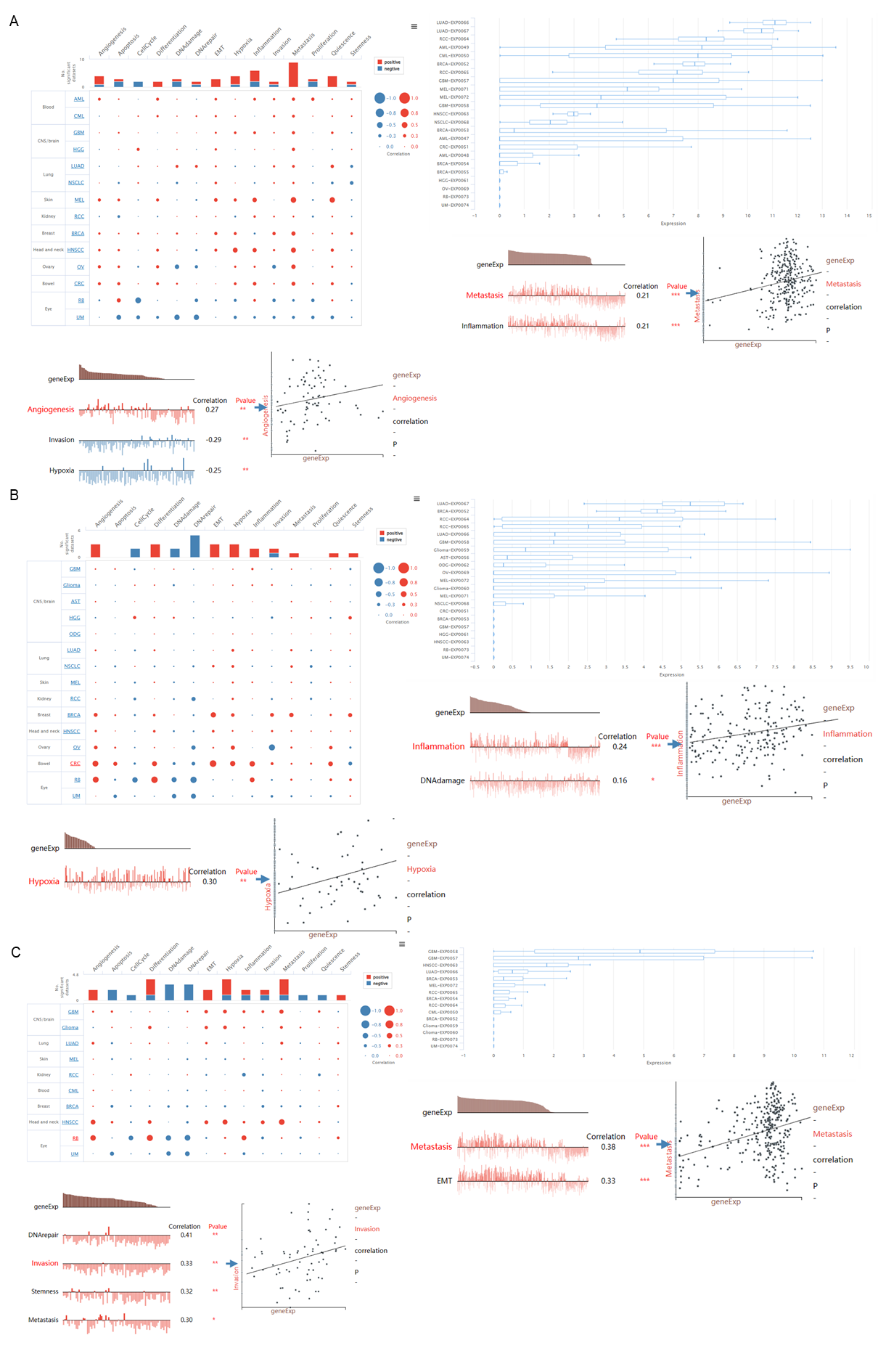

Supplement: Supplementary file 1 — Data S1. [file CAM4-12-17445-s001.zip › cam46316-sup-0007-FigureS7.tif]

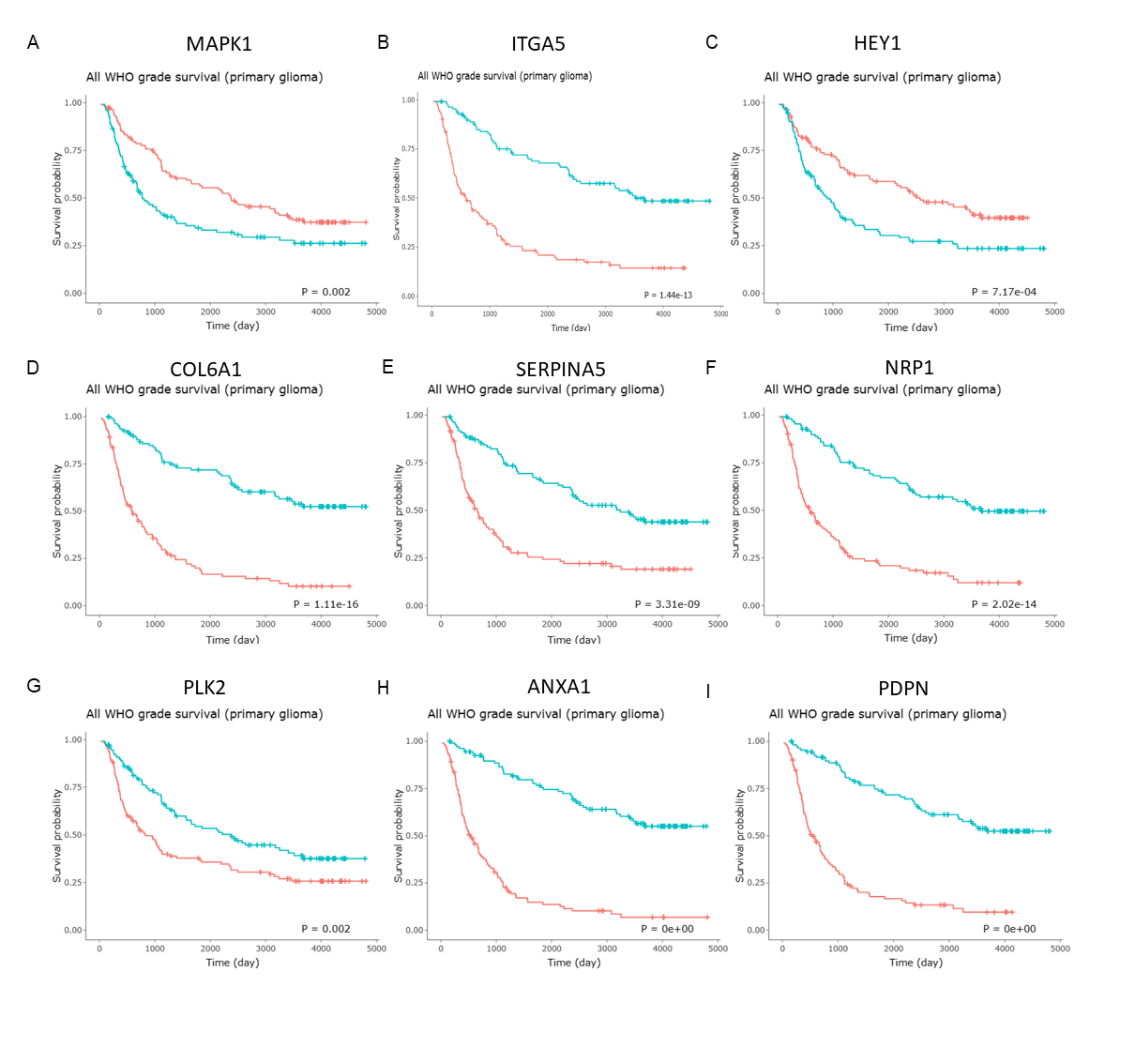

Supplement: Supplementary file 1 — Data S1. [file CAM4-12-17445-s001.zip › cam46316-sup-0008-FigureS8.tif]
